# Nanophotonic Materials and Devices: Recent Advances and Emerging Applications

**DOI:** 10.3390/mi16080933

**Published:** 2025-08-13

**Authors:** Yuan-Fong Chou Chau

**Affiliations:** Centre for Advanced Material and Energy Sciences, Universiti Brunei Darussalam, Tungku Link, Gadong BE1410, Brunei; chou.fong@ubd.edu.bn

**Keywords:** nanophotonics, light–matter interactions, plasmonic metals, two-dimensional (2D) materials, localized surface plasmon resonances, quantum-enhanced sensing

## Abstract

Nanophotonics, the study of light–matter interactions at the nanometer scale, has emerged as a transformative field that bridges photonics and nanotechnology. Using engineered nanomaterials—including plasmonic metals, high-index dielectrics, two-dimensional (2D) materials, and hybrid systems—nanophotonics enables light manipulation beyond the diffraction limit, unlocking novel applications in sensing, imaging, and quantum technologies. This review provides a comprehensive overview of recent advances (post-2020) in nanophotonic materials, fabrication methods, and their cutting-edge applications. We first discuss the fundamental principles governing nanophotonic phenomena, such as localized surface plasmon resonances (LSPRs), Mie resonances, and exciton–polariton coupling, highlighting their roles in enhancing light–matter interactions. Next, we examine state-of-the-art fabrication techniques, including top-down (e.g., electron beam lithography and nanoimprinting) and bottom-up (e.g., chemical vapor deposition and colloidal synthesis) approaches, as well as hybrid strategies that combine scalability with nanoscale precision. We then explore emerging applications across diverse domains: quantum photonics (single-photon sources, entangled light generation), biosensing (ultrasensitive detection of viruses and biomarkers), nonlinear optics (high-harmonic generation and wave mixing), and integrated photonic circuits. Special attention is given to active and tunable nanophotonic systems, such as reconfigurable metasurfaces and hybrid graphene–dielectric devices. Despite rapid progress, challenges remain, including optical losses, thermal management, and scalable integration. We conclude by outlining future directions, such as machine learning-assisted design, programmable photonics, and quantum-enhanced sensing, and offering insights into the next generation of nanophotonic technologies. This review serves as a timely resource for researchers in photonics, materials science, and nanotechnology.

## 1. Introduction

The manipulation of light at subwavelength scales has revolutionized photonics, allowing unprecedented control over electromagnetic fields for applications ranging from high-speed communications to quantum computing [1,2,3]. Nanophotonics, which explores light–matter interactions on the nanometer scale, has emerged as a key enabler of these advancements, overcoming the diffraction limit through engineered materials and nanostructures [4,5,6]. In classical optics, the diffraction limit (approximately λ/2 for visible light) sets a fundamental bound on the smallest spot size or feature that can be resolved with far-field beams. Nanophotonic structures bypass this limit by operating in the near-field regime, where evanescent fields enable light confinement at dimensions well below the free-space diffraction limit. By harnessing these near-field modes, nanophotonics can achieve subwavelength localization and manipulation of light that far-field optical elements cannot attain. During the past decade, the field has transitioned from fundamental discoveries to practical implementations, driven by innovations in material platforms, nanofabrication, and theoretical modeling [7,8,9,10,11,12].

The unique optical properties of nanophotonic materials arise from their ability to confine and enhance electromagnetic fields at deep subwavelength scales [13]. Plasmonic nanostructures, for example, exploit localized surface plasmon resonances (LSPRs) to generate intense near-field enhancements, while high-index dielectric nanoparticles leverage Mie resonances for low-loss light manipulation [14,15]. Two-dimensional (2D) materials, such as graphene and transition metal dichalcogenides (TMDCs), offer tunable optoelectronic properties, and hybrid systems combine these advantages to achieve multifunctional responses [16]. These materials form the foundation for devices such as ultracompact sensors, high-efficiency light emitters, and reconfigurable metasurfaces [17,18,19].

Recent years have witnessed remarkable progress in fabrication techniques that allow the precise synthesis and patterning of nanophotonic structures [20,21]. Top-down methods, such as electron beam lithography (EBL) and focused ion beam (FIB) milling, provide nanometer-scale accuracy, while bottom-up approaches, including chemical vapor deposition (CVD) and self-assembly, offer scalable and cost-effective alternatives [22,23]. Hybrid strategies, which integrate top-down patterning with bottom-up material growth, are increasingly adopted to balance precision with large-area manufacturability [24,25]. These advances have facilitated the development of wafer-scale nanophotonic devices, including metasurface optics and photonic integrated circuits (PICs) [26,27,28].

Nanophotonics applications span a broad spectrum of fields. In quantum photonics, nanophotonic cavities and waveguides enable the efficient generation and manipulation of single photons, critical for quantum communication and computing [29]. In biosensing, plasmonic and dielectric nanostructures have achieved attomolar detection limits for biomarkers and pathogens, paving the way for point-of-care diagnostics [30,31]. Nonlinear optical effects, such as harmonic generation and wave mixing, have been enhanced by using hybrid metasurfaces, enabling compact frequency converters and ultrafast optical switches. Meanwhile, the integration of nanophotonic components into PICs promises to transform data centers, telecommunications, and on-chip optical processing [32,33].

Despite these successes, there remain challenges. Optical losses in plasmonic materials, fabrication tolerances in dielectric resonators, and the environmental stability of 2D materials pose significant hurdles. Furthermore, the integration of nanophotonic devices with existing electronic and photonic platforms requires further innovation in heterogeneous fabrication and packaging [34]. To address these challenges, researchers are turning to advanced design tools, such as machine learning (ML)-assisted inverse design and physics-informed neural networks (PINNs), which accelerate the optimization of complex nanophotonic systems [35].

By synthesizing recent breakthroughs and identifying open questions [36], this review aims to guide researchers through the rapidly evolving landscape of nanophotonics [37,38] and inspire future innovations in this dynamic field [39]. A key focus is the development of engineered nanomaterials, where advances in nanofabrication, material synthesis, and optical characterization enable precise control over refractive indices, resonances, and electromagnetic responses [40]. These material platforms—including plasmonic nanostructures, low-loss dielectric nanoparticles, 2D materials (e.g., graphene and TMDCs), and hybrid systems—underpin advanced devices such as metasurface lenses, compact modulators, photonic crystal cavities, and quantum emitters, offering superior performance and integration potential [41,42].

In this review, five representative domains of nanophotonic applications were selected to encompass the spectrum from fundamental phenomena to real-world implementations. Quantum photonics and nonlinear optics are included as they represent fundamental photonics research areas where nanostructures enable new physics (e.g., single-photon generation and ultrafast frequency conversion). Biosensing is highlighted as a key applied domain in which nanophotonics has had a significant impact on technology (ultrasensitive detection and diagnostics). Photonic integrated circuits (PICs) and metasurfaces are featured as versatile physical platforms that translate nanoscale optical effects into device-level applications—PICs exemplify on-chip integration of nanophotonic components, while metasurfaces demonstrate planar optical devices replacing bulk optics. Together, these categories were chosen to provide a coherent overview of the nanophotonics landscape, linking basic research to enabling platforms and end-use technologies.

We provide a comprehensive overview of nanophotonic materials and their emerging applications. Section 2 discusses the types of materials and underlying principles, while Section 3 covers fabrication methods, from top-down and bottom-up approaches to hybrid strategies. Section 4 explores applications in quantum photonics, biosensing, nonlinear optics, integrated photonics, and metasurfaces. Section 5 highlights key advances, including active tunable devices, large-area fabrication, hybrid integration, and AI-designed photonics. Section 6 addresses remaining challenges, Section 7 provides a future outlook on machine learning-guided design, low-loss materials, programmable metasurfaces, and quantum-enhanced nanophotonics, pointing toward future opportunities in this dynamic field, and Section 8 presents the conclusion.

## 2. Core Principles of Nanophotonics-Based Nanomaterials

Nanophotonic materials enable subwavelength light manipulation through tailored interactions with electromagnetic fields. This section reviews four key material classes, plasmonic, dielectric, 2D, and hybrid systems, highlighting their physical principles, recent advances, and comparative performance [43].

### 2.1. Plasmonic Nanomaterials

Plasmonic materials (e.g., Au, Ag, Al) support localized surface plasmon resonances (LSPRs) and propagating surface plasmon polaritons (SPPs), facilitating intense field confinement. The resonance condition is governed by the following.

$$\omega_{LSPR}=\frac{\omega_{P}}{\sqrt{2\varepsilon_{\infty }+\varepsilon_{m}}}$$

where ω_p_ is the plasma frequency, $\varepsilon_{\infty }$ is the high-frequency permittivity, and m means the dielectric environment (that is, the dielectric constant of the surrounding medium).

The resonance frequency, determined by the collective oscillation of the conduction electrons, is highly sensitive to the surrounding dielectric environment. Recent advances have introduced low-loss alternatives such as titanium nitride (TiN) and indium tin oxide (ITO), which combine plasmonic performance with CMOS compatibility [44]. Quantum plasmonics has further pushed boundaries, enabling single-molecule detection via surface-enhanced Raman spectroscopy (SERS) with unprecedented sensitivity [45]. Despite these breakthroughs, challenges such as ohmic losses persist, driving research into non-local effects and hybrid systems incorporating gain materials. Plasmonic nanostructures are now widely used in biosensing (exemplified by their ability to detect viral proteins at attomolar concentrations) and in energy applications, where they improve photocatalytic hydrogen generation [46,47].

#### Plasmonic Nanostructures

In modern nanophotonic devices, various plasmonic nanostructures serve as fundamental building blocks for field enhancement and light manipulation. For example, C-apertures (C-shaped nanoapertures in metal films) act as polarization sensitive antennas that concentrate optical near-fields into deep subwavelength volumes [48,49]. Bowtie nanoantennas, consisting of opposing triangular metal tips separated by a nanogap, produce extremely high local field intensities at the gap, enabling single-molecule surface-enhanced Raman sensing and nano-optical trapping [50]. Arrays of plasmonic nanopillars (nanoscale metal or metal coated pillars) support localized surface plasmons along their shafts or tips and are widely used to create high-density ‘hot spots’ for enhanced spectroscopy and light emission control [51,52]. Similarly, zero-mode waveguides, subwavelength circular apertures in opaque metal films, confine light to attoliter-scale volumes by cutting off propagating modes, and have revolutionized single-molecule fluorescence detection beyond the diffraction limit [53]. Each of these plasmonic nanostructures offers a unique mechanism for concentrating and controlling light at the nanoscale.

### 2.2. Dielectric Nanomaterials

Dielectric nanomaterials, including silicon and titanium dioxide, utilize Mie resonances to achieve low-loss light confinement. The electric (a_n_) and magnetic (b_n_) Mie coefficients (which represent the amplitude of the scattered electromagnetic waves) are:

(1) $$\alpha_{n}=\frac{m\Psi_{n}\left(mx\right)\Psi_{n}^{\prime }\left(mx\right)-\Psi_{n}\left(x\right)\Psi_{n}^{\prime }\left(mx\right)}{m\Psi_{n}\left(mx\right)\xi_{n}^{\prime }\left(mx\right)-\xi_{n}\left(x\right)\Psi_{n}^{\prime }\left(mx\right)}$$

(2) $$b_{n}=\frac{\Psi_{n}\left(mx\right)\Psi_{n}^{\prime }\left(x\right)-m\Psi_{n}\left(x\right)\Psi_{n}^{\prime }\left(mx\right)}{\Psi_{n}\left(mx\right)\xi_{n}^{\prime }\left(x\right)-m\xi_{n}\left(x\right)\Psi_{n}^{\prime }\left(mx\right)}$$

where m is the refractive index contrast (m = n_partical_/n_medium_), and x is the size parameter (x = $\frac{2\pi rn_{medium}}{\lambda }$). r is the radius of the particle radius and λ is the wavelength. ψ_n_(z) and ξ_n_(z) denote Riccati–Bessel functions (related to spherical Bessel functions), ψ_n_(z) = zj_n_(z) (regular Riccati–Bessel function) and ξ_n_(z) = zh_n_^(1)^(z) (outgoing spherical Hankel function). High-index dielectrics (Si, TiO_2_, GaP) leverage Mie resonances for low-loss light control.

These materials support both electric and magnetic multipolar resonances, allowing for advanced optical functionalities. Recent developments in all-dielectric metasurfaces have led to ultraefficient lenses capable of diffraction-limited focusing, while nonlinear effects in materials like gallium phosphide enable significant enhancements in harmonic generation. The compatibility of dielectric nanostructures with semiconductor fabrication techniques makes them ideal for scalable photonic integration. Emerging trends, such as topological photonics, further expand their potential by creating robust optical states that are immune to structural disorder. Key developments include (1) all-dielectric metasurfaces: TiO_2_ metalenses achieving diffraction-limited focusing and (2) nonlinear optics: GaP nanoparticles that enhance third-harmonic generation (THG) by over 10^6^× [54]. The advantages of dielectric nanomaterials include (i) low absorption (e.g., α < 1 dB/cm for Si at 1550 nm) [55] and (ii) compatibility with CMOS processes [56].

### 2.3. Two-Dimensional (2D) Materials

Two-dimensional materials, such as graphene and transition metal dichalcogenides (TMDCs), offer tunability and strong excitonic effects [57]. Graphene gate-tunable plasmons provide dynamic control over light at terahertz frequencies, paving the way for high-speed optical modulators. Meanwhile, TMDCs such as molybdenum disulfide (MoS_2_) exhibit valley-selective photoluminescence, opening new avenues for valleytronics. Recent progress includes the integration of graphene-based modulators into silicon photonic circuits for next-generation communications and the discovery of room-temperature single-photon emitters in hexagonal boron nitride (hBN). However, environmental instability remains a critical challenge, requiring the development of advanced encapsulation techniques [58,59].

These fundamental properties have enabled practical advances; for example, using graphene tunable plasmons, researchers have integrated graphene-based modulators into silicon photonic circuits for high-speed optical communication, and the strong excitonic emission of hBN has led to the realization of room-temperature single-photon sources. (These examples are detailed further in Section 4).

### 2.4. Hybrid Nanophotonic Systems

Hybrid nanophotonic systems combine the strengths of plasmonic, dielectric, and 2D materials to achieve multifunctional performance. For example, graphene–plasmonic hybrids enable electrically tunable absorbers with near-perfect modulation depth, while quantum dot–metasurface systems enhance single-photon emission for quantum technologies [60,61]. Phase-change materials like Ge_2_Sb_2_Te_5_ (GST) have been integrated into hybrid platforms to create reconfigurable optical devices with nonvolatile switching capabilities.

Plasmonic materials excel in applications requiring intense field confinement (e.g., biosensing) but suffer from optical losses. Dielectrics offer low-loss solutions for meta-optics but lack dynamic tunability. Two-dimensional materials bridge this gap with their atomic-scale tunability, while hybrid systems unlock unprecedented functionality by combining these advantages [62].

Hybrid systems harness multiple material platforms simultaneously (e.g., graphene–plasmonic absorbers or quantum dot–dielectric metasurfaces) to achieve functionalities unattainable by a single material alone. (Detailed examples of hybrid system applications are provided in Section 4. Detailed strategies for nanophotonic design optimization, including emerging machine learning-assisted approaches, are discussed in Section 5).

Table 1 presents a comprehensive comparison of key nanophotonic material platforms, including plasmonics, dielectrics, 2D materials, and hybrid systems (such as plasmonic–dielectric combinations). This table evaluates their optical properties including loss, tunability and the example applications.

**Figure 1:**
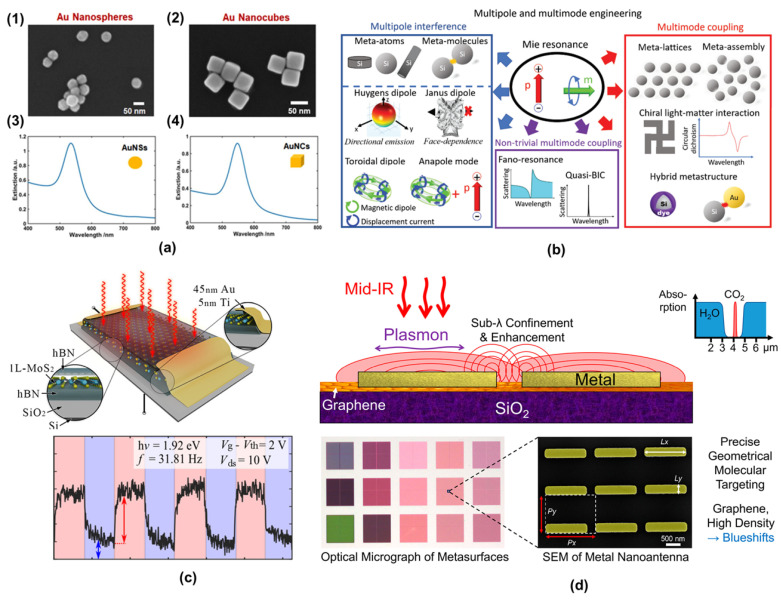
Schematic of light–matter interactions in nanophotonic materials, including (**a**) LSPR in gold nanoparticles, (**b**) Mie resonances in silicon nanospheres [63], (**c**) excitons in MoS_2_ monolayers [65], and (**d**) a hybrid graphene–silicon metasurface [64]. Panels (**a**–**d**) are reproduced with permission from open-access articles (refs. [63,64,65]).

To visualize these principles, Figure 1 illustrates key light–matter interactions across material classes, including (a) an LSPR in gold nanoparticles, (b) Mie resonances in silicon nanospheres [63], (c) excitons in MoS_2_ monolayers, and (d) a hybrid graphene–silicon metasurface [64]. These examples underscore the diversity and potential of nanophotonic materials in the advancement of technologies ranging from quantum computing to ultrasensitive diagnostics.

## 3. Fabrication Techniques

The realization of functional nanophotonic devices is critical to the advancement of advanced fabrication techniques capable of manipulating matter at the subwavelength scale. These methods have evolved significantly in recent years, driven by the need to balance nanometer-scale precision with scalable manufacturing [66]. This section provides an in-depth examination of contemporary fabrication paradigms, highlighting their physical principles, technological innovations, and application-specific advantages [67]. Nanophotonic structure fabrication requires precise patterning and control over material properties at the nanoscale. Techniques are broadly classified into top-down, bottom-up, and emerging hybrid approaches [68]. Each approach offers distinct advantages in terms of resolution, throughput, and material versatility, and recent innovations continue to push the limits of device complexity and scale [69,70].

### 3.1. Top-Down Approaches

Top-down methods create nanostructures by starting from bulk materials and removing or shaping material using lithography and etching. These techniques offer nanometer-scale resolution and pattern accuracy [71,72]. Key top-down fabrication methods include

(1) Electron beam lithography (EBL): EBL uses a focused electron beam to write patterns directly into a resist film on a substrate. It is widely used for prototyping nanophotonic structures due to its superb resolution (feature sizes < 10 nm are achievable). Complex shapes such as photonic crystal lattices or metasurface patterns can be precisely defined. However, EBL is a serial process (writing patterns point-by-point) and thus is time-consuming and expensive for large areas. It is most suitable for research-scale fabrication or mask manufacturing but not for high-volume manufacturing.
(2) Focused ion beam (FIB) milling: FIB directs a focused beam of ions (commonly Ga^+^ ions) to sputter material from a surface, effectively ‘carving’ nanostructures. FIBs can also be used to deposit materials via ion-beam-induced deposition and provide great flexibility in 2D and shallow 3D patterning (for example, drilling nanopores or waveguide facets) with resolution on the order of a few tens of nanometers. FIB is often used for rapid prototyping, mask repair, or post-fabrication trimming of photonic devices; although like EBL, it is slow and not easily scalable to large wafer areas [73]. However, one limitation of Ga^+^-based FIB is ion implantation: the gallium ions can become embedded in the patterned material, leading to contamination and defects in nanophotonic structures [74]. This phenomenon is known to alter optical properties (e.g., increasing loss or changing refractive index) in sensitive devices. To address this, researchers have explored alternate milling sources (such as He^+^ or Ne^+^ ion beams) and post-fabrication treatments to reduce Ga-induced damage [75,76,77].
(3) Photolithography: Photolithography uses ultraviolet (UV) or deep-UV light and masks to pattern large areas in parallel. Modern photolithography (including 193 nm immersion lithography and extreme ultraviolet (UV) lithography at 13.5 nm wavelength) can produce features below 50 nm on entire wafers with high throughput, using the same processes that drive semiconductor chip production. Although photolithography is extremely scalable and fast for repeated patterns, it typically requires expensive masks and is less accessible for rapid iteration compared to EBL. It is commonly used for mass-manufacturing integrated photonic circuits (e.g., silicon photonics) where feature sizes are within the resolution limits.
(4) Nanoimprint lithography (NIL): Nanoimprint is a technique that bridges some advantages of top-down and replication methods. In NIL, a hard mold (stamp) containing nanostructures is pressed into a resin on a substrate, physically imprinting the nanopattern, which is then cured (by heat or UV light) and lifted off. NIL can achieve extremely high resolution (down to ~10 nm features) and, being a parallel process, can pattern large areas rapidly once a master mold is made. It has emerged as a promising method for the high-throughput fabrication of metasurfaces and other nanophotonic devices. The challenge in NIL lies in making a defect-free mold and avoiding defects during the imprint step (e.g., due to the sticking of the particles or resist sticking). However, roll-to-roll nanoimprint processes have been developed for flexible substrates, pointing toward industrial-scale nanophotonics manufacturing [78].
(5) Bilayer resist lithography: Bilayer resist lithography is an advanced top-down approach now mentioned in our review. In this technique, two different resist layers (with different sensitivities or etch characteristics) are used during EBL to achieve enhanced patterning outcomes. For instance, a bilayer EBL process can create a reentrant or undercut resist profile, enabling clean lift-off of nanostructures with very fine features, or it can improve the aspect ratio by using a sturdier underlayer as an etch mask. We have included a brief description of this method and its utility in the definition of high-resolution nanophotonic structures [79,80]. Additionally, we have introduced template stripping as a fabrication technique. Template stripping involves fabricating nanostructures on a super-smooth template substrate and then bonding and peeling off a target substrate to transfer the nanostructures. This method produces extremely smooth metallic nanostructures (inherited from the atomic flatness of the template) and has been used to produce low-loss plasmonic surfaces and nanohole arrays at the wafer scale [81,82,83]. By incorporating bilayer lithography and template stripping into our discussion, we acknowledge these important techniques and the role they play in pushing the limits of nanofabrication for photonic devices.

Top-down approaches, especially EBL and photolithography, remain indispensable for fabricating the fine features required in nanophotonics. They offer excellent control over geometry, which is critical for devices such as photonic crystal cavities or plasmonic antennas, where performance is sensitive to nanoscale dimensions. The primary trade-off is throughput and cost [84]. Research efforts are ongoing to improve the speed of EBL (for example, through multibeam electron lithography) and to develop maskless optical lithography techniques that bypass expensive mask fabrication [85]. Moreover, advanced etching techniques (reactive ion etching, ion beam etching) are used after lithography to transfer patterns into different materials (silicon, III–V semiconductors, metals) with vertical sidewalls and smooth surfaces necessary for low optical loss [86].

### 3.2. Bottom-Up Approaches

Bottom-up methods rely on chemical synthesis and self-assembly to construct nanostructures from molecular or atomic precursors. Instead of carving structures from bulk material, bottom-up fabrication builds structures by assembling smaller building blocks [87,88]. Key bottom-up techniques relevant to nanophotonics include:

(1) Chemical Synthesis of Nanomaterials: A diverse range of nanostructures can be synthesized in solution with precise control over their size, shape, and composition. Common examples include colloidal semiconductor quantum dots, metallic nanoparticles (such as nanospheres, nanorods, and nanostars), and dielectric nanocrystals. This solution-based approach enables tunable optical and electronic properties, making it highly valuable for applications in photonics, catalysis, and biomedical imaging. These solution-grown nanomaterials can exhibit the desired optical resonances (e.g., plasmonic or excitonic peaks) and can be subsequently deposited or arranged on surfaces for device integration. Wet chemical synthesis is relatively low-cost and can produce nanomaterials in large quantities, but arranging these colloids into functional devices often requires additional steps [89].
(2) Chemical Vapor Deposition (CVD): CVD and related epitaxial growth techniques can produce thin films and nanostructures on substrates. For instance, large-area graphene and TMDC monolayers are commonly grown by CVD on catalytic substrates. Similarly, semiconductor nanowires can be grown by vapor–liquid–solid epitaxy. These as-grown materials often form the basis of nanophotonic devices (e.g., a monolayer MoS_2_ integrated into a photonic cavity). The bottom-up CVD approach excels in producing high-crystallinity materials and novel heterostructures (such as vertically stacked 2D materials), although placement and patterning must be achieved by templating the growth or by post-growth lithography [90,91].
(3) Self-Assembly and Template-Assisted Assembly: In self-assembly, components (such as nanoparticles, block copolymers, or molecules) spontaneously organize into ordered structures driven by thermodynamics. For nanophotonics, self-assembly can create periodic nanoparticle arrays, photonic crystals, or metamaterial structures over large areas without expensive tools. For example, colloidal nanospheres can self-assemble into 2D or 3D periodic lattices (acting as natural photonic crystals), and block-copolymer lithography can produce dense periodic patterns at the sub-20 nm scale by phase separation of polymers. However, purely self-assembled structures may lack the long-range order or registration needed for complex circuits. Template-assisted self-assembly combines top-down and bottom-up: lithographically defined templates (like shallow topographical patterns or chemically functionalized sites on a substrate) guide the assembly of nanomaterials into predetermined locations and arrangements. This hybrid strategy can produce ordered nanostructures in large areas by using chemical forces to conduct placement, thus marrying precision with scalability.
(4) Bottom-up approaches offer cost-effective fabrication over large areas and access to material systems that may be difficult to pattern with lithography (for example, growing crystalline materials with atomic precision or creating nanostructures in a chemical beaker) [92]. The trade-off is that achieving arbitrary or complex patterns is challenging; self-assembled structures are often limited to relatively simple or periodic patterns, and positioning of specific features (like a single quantum dot in a cavity) can be stochastic. However, bottom-up fabrication is essential to build the materials foundation of nanophotonics (such as single-crystal nanomaterials or quantum emitters) and is often combined with top-down patterning for device integration [93].

### 3.3. Hybrid Fabrication Strategies

Hybrid strategies combine the strengths of top-down and bottom-up approaches to overcome their individual limitations. The goal is to achieve high resolution and precision, and scalability and cost-efficiency in a fabrication workflow [94]. Several hybrid fabrication paradigms have emerged:

(1) Template-Guided Self-Assembly: As noted above, a lithographically defined template can be used to direct bottom-up growth or assembly. For example, electron beam lithography can define an array of nanoscale trenches or chemically modified sites on a substrate, and solution-assisted colloidal nanoparticles (or nanowires, etc.) can preferentially attach to these sites [95]. This results in an ordered array of nanoparticles aligned with the template pattern. Such approaches have been used to fabricate plasmonic nanoparticle arrays and even DNA-directed assembly of quantum dots on photonic chips, achieving placement accuracy better than random self-assembly.
(2) Nanoimprint and Transfer Techniques: Nanoimprint lithography itself is a hybrid method—the mold is typically made by top-down means (EBL, for example) but once created, it can be used repetitively as a stamp to replicate patterns in a bottom-up fashion. Another hybrid concept is transfer printing: nanostructures (such as nanowires, 2D material flakes or prefabricated nanopillars) can be fabricated or grown on one substrate and then transferred and embedded on a target photonic chip using polymer stamps or adhesion layers [96]. This enables the integration of materials that are incompatible with standard lithography (for example, placing a single-crystal nanowire laser on a silicon photonic waveguide) [97].
(3) Direct laser writing (multiphoton lithography): Advances in additive manufacturing allow true 3D nano- and micro-fabrication via two-photon polymerization. In this technique, a focused femtosecond laser causes localized polymerization in a photosensitive resin at the focal point, which can be scanned in 3D to ‘print’ arbitrary 3D microstructures such as photonic woodpiles, microlenses, or even complex free-form meta-optics. Direct two-photon laser writing is a maskless technique that combines top-down control (the laser focus defines the pattern) with a bottom-up building of structure. The resolution can reach ~100 nm. While currently serial and relatively slow, it offers design geometries not achievable by planar lithography and has been used to create 3D photonic crystals and gradient-index micro-optics for nanophotonics.
(4) Laser-Assisted Fabrication and Growth: Techniques such as laser interference lithography (which uses laser interference patterns to expose periodic patterns on a resist) and laser annealing can rapidly produce large-area periodic nanostructures or activate material growth [98]. For example, interference lithography can create a photonic crystal pattern in one step over a wafer, and pulsed laser deposition can create non-equilibrium material films with unique optical properties. These methods are hybrids in the sense that they leverage optical fields (bottom-up in effect) to drive top-down patterning.

Selecting a fabrication methodology is critically on the target application for resolution, throughput, and material compatibility. Although top-down approaches remain essential for prototyping and certain high-performance devices, hybrid strategies are increasingly being adopted in commercial applications requiring both precision and scalability. The ongoing convergence of nanofabrication techniques with computational design tools and machine learning is expected to further blur the boundaries between these paradigms, enabling the creation of increasingly complex nanophotonic systems [99].

By intelligently combining approaches, hybrid fabrication schemes are achieving complex, high-performance nanophotonic devices at a scale. A notable example is metasurface fabrication: one strategy uses deep-UV or nano-imprint lithography to create an initial metasurface pattern and then employs self-aligned etching or deposition to fine-tune nanoscale features, achieving both large-area coverage and nanoscale precision [100]. Another example is integrating active materials (like quantum dots, organic molecules, or phase-change materials) into nanophotonic structures: top-down lithography defines the device, and bottom-up processes embed the active material (e.g., spin-coating quantum dots into a nanoantenna array or using CVD to grow a 2D layer onto a nanocavity). Such multistep processes are becoming more common as nanophotonic systems increase in functionality [101].

NIL exemplifies a top-down hybrid approach: a nanopatterned mold can be used to replicate nanostructures in a bottom-up fashion onto large substrates, bypassing the diffraction limit of light and enabling high-throughput nanofabrication.

In general, the continued development of advanced fabrication techniques is a driving force in nanophotonics. Innovations such as multibeam lithography, nanoscale 3D printing, and roll-to-roll nanomanufacturing are expected to further push the boundaries, making it feasible to produce complex nanophotonic devices in a cost-effective manner at industrial volumes. These advances will be crucial for the transition of nanophotonic technologies from laboratory demonstrations to real-world applications [85]. Table 2 summarizes the main fabrication approaches, highlighting their advantages and limitations in the context of nanophotonics.

Section 4 discusses key domains of nanophotonics and recent advances in each.

## 4. Applications

The transformative potential of nanophotonic materials is being realized in an expanding range of cutting-edge applications. By engineering light–matter interactions at subwavelength scales, these advanced materials are enabling breakthroughs that were previously restricted by the diffraction limit of conventional optics [102]. This section examines five key application domains where nanophotonics is making significant impacts, with a detailed technical analysis of recent advances and future prospects.

### 4.1. Quantum Photonics

In quantum photonics, nanophotonic materials are essential for manipulating single photons and entangled quantum states of light. Achieving strong light confinement in nanostructures is key to improving interactions involving single quantum emitters or nonlinear processes at the single-photon level [103]. Two main thrusts in quantum photonics where nanomaterials have significant impact are single-photon sources and quantum information processing on chip [104].

Nanophotonics can greatly improve single-photon sources by enhancing emission rates and extraction efficiency. For example, plasmonic and dielectric nanoantennas can enhance spontaneous emission rates via the Purcell effect, resulting in bright and fast single-photon emitters [105]. The Purcell effect is the enhancement of spontaneous emission that occurs when the emitter is placed in a resonant optical environment with a high local density of photonic states. Nanophotonic cavities or antennas with small mode volumes and high Q-factors can greatly increase emission rates by this mechanism. Photonic crystal cavities with ultrasmall mode volumes and high-quality factors provide tailored electromagnetic environments for emitters, enabling phenomena such as cavity quantum electrodynamics (strong coupling between a single emitter and a single-photon mode). Hybrid nanostructures allow integration of quantum emitters (such as semiconductor quantum dots, atoms, or color centers in diamonds) with nanophotonic circuits. A notable achievement was the integration of a single solid-state defect (an ‘atom-like’ quantum emitter in diamond) into a nanophotonic cavity, which produced streams of high-purity single photons in a fully chip-integrated system. This demonstrates a single-photon on-demand source suitable for quantum information processing, with the nanocavity providing the necessary optical enhancement and scalability for integration [106,107].

Another area is the generation and manipulation of entangled photons and quantum states on photonic chips [108]. Waveguide-integrated nonlinear nanomaterials (like silicon nitride microring resonators or periodically poled lithium niobate waveguides) can generate entangled photon pairs via processes such as spontaneous parametric down-conversion or four-wave mixing. Nanophotonic circuits (using beam splitters, phase shifters, and single-photon detectors integrated on the same chip) are being developed to perform quantum logic operations and quantum interference with high stability [109]. Chip-scale integration has become critical for scaling up quantum photonic systems beyond tabletop experiments. Indeed, as in classical photonics, the drive is toward quantum photonic integrated circuits (QPICs) that monolithically or hybridly integrate sources, detectors, and linear optical elements. Recent roadmaps highlight that integrated photonics bring robustness and scalability to quantum technologies and are expected to enable applications such as photonic quantum computing and secure quantum communication networks [110]. For instance, significant milestones such as achieving quantum computational advantage with photonic circuits have been reported (e.g., demonstration of photonic ‘quantum supremacy’ through Gaussian boson sampling in 2020), underscoring the progress in this field [111,112].

**Figure 2:**
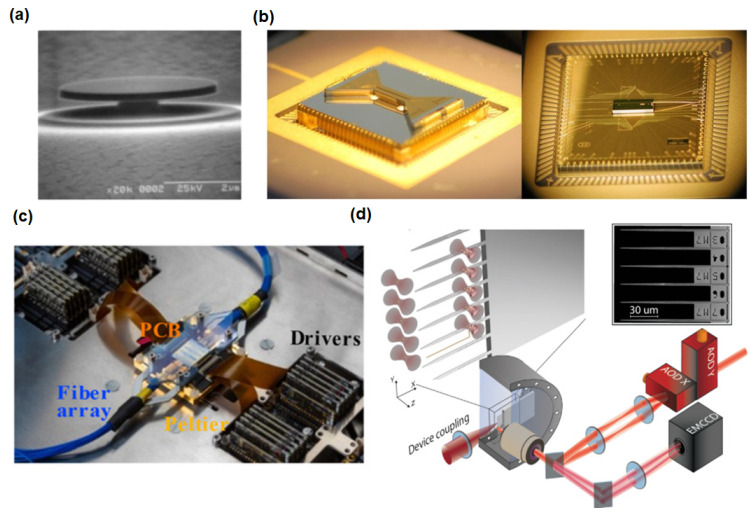
A representative nanophotonic quantum photonic system. (**a**) Single-photon source: a microdisk resonator with whispering gallery modes (WGMs) couples to a single InAs quantum dot, enhancing spontaneous emission (SE). [114] (**b**) On-chip routing: advanced microfabricated ion traps. LEFT: High-optical access (HOA) trap from Sandia National Laboratories (image courtesy of Duke University). RIGHT: Ball-grid array (BGA) trap from GTRI/Honeywell (image courtesy of Honeywell) [115]. (**c**) Reconfigurable coupling: picture of the photonic assembly of the 12-modes processor as mounted inside the control box [116]. (**d**) Integrated detection: optical tweezer array (Cs atoms) coupled to on-chip nanophotonic devices (SEM inset). Key components: AOD, EMCCD, and ultrahigh vacuum chamber [117]. Panels (**a**–**d**) are reproduced with permission from open-access articles (refs. [114,115,116,117]).

Nanophotonic structures also facilitate quantum–classical interfacing. They can route single photons in complex networks, enhance nonlinear interactions for single-photon frequency conversion, or even mediate interactions between solid-state qubits (such as NV centers or quantum dots) and photonic flying qubits (single photons) for quantum repeaters [113]. Looking ahead, combining quantum emitters (quantum dots, 2D material single-photon emitters, etc.) with metasurfaces and nanocavities could lead to ultracompact quantum light sources and sensors. In summary, nanophotonics provides the enabling toolkit for quantum photonics by offering the control and integration needed to build scalable chip-based quantum devices. Figure 2 shows a representative nanophotonic quantum photonic system, i.e., (a) single-photon source: a microdisk resonator with whispering gallery modes (WGMs) couples to a single InAs quantum dot, enhancing spontaneous emission (SE), (b) on-chip routing: a silicon nitride photonic circuit (gray) routes wavelength-multiplexed lasers (colored waveguides) to trapped ions (yellow: waveguide splitting zone; modulators not shown), (c) reconfigurable coupling: a 12-mode photonic processor chip (16 × 22 mm) for tunable quantum state manipulation, and (d) integrated detection: optical tweezer array (Cs atoms) coupled to on-chip nanophotonic devices (SEM inset). Key components: AOD, EMCCD, and ultrahigh vacuum chamber.

### 4.2. Biosensing

Plasmonic and dielectric nanostructures offer ultrasensitive platforms for biosensing, capable of detecting low concentrations of biomolecules via enhanced light–matter interactions. In these sensors, the presence of a biological analyte (such as a protein, DNA, virus particle, or cell) causes a measurable change in an optical signal (such as a resonance shift or intensity change). Nanophotonic materials improve both the sensitivity and compactness of biosensors [118].

One widely exploited mechanism is localized surface plasmon resonance (LSPR) in metallic nanostructures. LSPR biosensors (e.g., arrays of gold nanoparticles or nanoholes) provide label-free detection: the target molecules binding near the metal surface change the local refractive index, changing the plasmon resonance wavelength [14]. Due to the extreme concentration of the optical field at the nanoparticle surface—where enhancement and localization occur on the scale of tens of nanometers—even the binding of a small number of molecules can induce a measurable spectral shift. This enables the detection of analytes at femtomolar concentrations or the observation of single binding events under the right conditions [119]. For example, plasmonic sensors have been developed for the real-time detection of viruses such as SARS-CoV-2. During the COVID-19 pandemic, nanophotonic biosensors have emerged as valuable diagnostic tools due to their rapid response and high sensitivity [120]. In one demonstration, an LSPR chip functionalized to capture SARS-CoV-2 antibodies achieved the detection of extremely low antibody concentrations, outperforming traditional ELISA in sensitivity. Such a sensor, based on gold nanorods coated with viral antigen, could identify the presence of antibodies in patient samples in minutes, highlighting the potential of plasmonic nanophotonics in point-of-care testing [121,122].

Dielectric nanophotonic structures are also employed in biosensing. Photonic crystal slabs and nanostructured waveguides can support high-Q resonances or guided-mode resonances that are sensitive to surface binding events [123]. For example, a silicon photonic microring resonator can act as a biosensor where the binding of biomolecules on its surface shifts its resonance frequency; arrays of these can be used for multiplexed detection of various biomarkers. Unlike plasmonic sensors, dielectric sensors can have very low loss and can be integrated with on-chip light sources and detectors, allowing fully integrated lab-on-chip platforms [124].

Another powerful nanophotonic biosensing approach is surface-enhanced Raman scattering (SERS). By engineering plasmonic ‘hot spots’ (regions of extremely high field intensity, such as nanoparticle junctions or crevices in metallic nanostructures), the Raman signals of molecules can be enhanced by factors of 10^6^ or greater due to plasmonic effects. SERS-based nanophotonic substrates allow the detection of molecular fingerprints at very low concentrations, which are useful for chemical sensing of toxins, drugs, or biomolecular markers. Recent work has combined SERS substrates with microfluidics to enable portable sensors for, e.g., rapid virus detection in a drop of blood or saliva [125].

Integration with microfluidics and CMOS electronics further enhances the capabilities of nanophotonic biosensors. Compact, chip-scale biosensor devices have been demonstrated to be able to analyze multiple analytes with small sample volumes. For instance, nanoplasmonic sensors integrated in a microfluidic flow cell can perform real-time monitoring of binding kinetics for drug discovery. Photonic integrated circuits incorporating interferometric or ring resonator sensors are being developed for applications such as wearable health monitors and environmental sensing [126].

**Figure 3:**
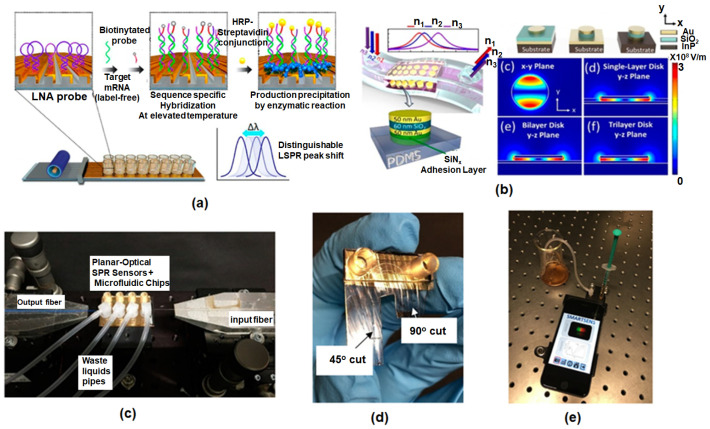
Schematic of a nanophotonic LSPR biosensor for real-time biomolecule detection on a microfluidic chip. The binding of target biomolecules to plasmonic nanostructures induces a measurable LSPR shift. (**a**) MiRNA detection with an inverted L-shaped nanostructure and nanograting-patterned substrate functionalized with locked nucleic acids (LNAs) [128]. (**b**) Cancer cell detection on a metal–insulator–metal (MIM) plasmonic structure integrated into a PDMS microfluidic channel [129]. (**c**) Experimental setup with an SPR sensor integrated into a microfluidic chip (center) and two optical fibers for light coupling (input on the right via a 45° cut, output on the left via a 90° cut with a diffraction grating) [130]. (**d**) Assembly of fiber coupling structures and microfluidic components within a 3D-printed enclosure [127]. (**e**) The sensor chip was placed into a 3D-printed housing [127]. Panels (**a**–**c**) reproduced with permission from Refs. [128,129,130]; panels (**d**,**e**) reproduced with permission from ref. [127].

Figure 3 illustrates a nanophotonic biosensor based on LSPR for real-time biomolecule detection within a microfluidic platform. Target analytes captured by plasmonic nanostructures inside the microchannel induce a quantifiable LSPR spectral shift. Microfluidic flow ensures controlled delivery to the sensing elements, allowing real-time analysis. Specific LSPR biosensing strategies shown in Figure 2 include: (i) miRNA detection using an inverted L-shaped nanostructure with nanograting patterns, functionalized with locked nucleic acids (LNA) [127]; (2) cancer cell detection on a metal–insulator–metal (MIM) structure integrated into PDMS, which captures cancer cells [127]; (3) an SPR/microfluidic setup with a central microfluidic chip interfacing with an SPR sensor. Light is coupled through a 45°–cut optical fiber (right) through total internal reflection and exits through a 90° cut fiber (left) using a reflection-mode diffraction grating [119]; and (4) assembly of coupling structures and microfluidic components, housed in a 3D-printed enclosure [119].

In summary, nanophotonics has significantly advanced biosensing by providing (1) enhanced sensitivity (through field confinement and amplification, as in LSPR and SERS), (2) label-free operation (detecting refractive index changes or vibrational signatures directly), and (3) potential for miniaturization into portable devices. Ongoing research addresses challenges such as sensor surface biofunctionalization, reducing nonspecific binding, and improving the reproducibility and cost of nanofabricated sensors. Given the progress to date, nanophotonic biosensors are poised to play an increasingly important role in healthcare (e.g., early disease diagnosis, pandemic monitoring), environmental monitoring, and food safety.

### 4.3. Nonlinear Optics

The enhanced electromagnetic field intensities in nanophotonic structures make them ideal platforms for nonlinear optical processes. Nonlinear optics involve phenomena where the response of a material to light is nonlinear in the light field strength, giving rise to effects such as frequency conversion (generation of new colors of light), optical modulation, and ultrafast signal processing. Nanostructures can greatly increase the efficiency of these processes by concentrating light in tiny volumes or using materials with large nonlinear coefficients [131].

Common nonlinear processes pursued in nanophotonics include second harmonic generation (SHG), third harmonic generation (THG), four-wave mixing (FWM), and Raman scattering, among others. For these effects, the efficiency typically scales with some power of the optical field intensity, so nanophotonic resonators and waveguides that amplify intensity can dramatically boost nonlinear signals. For example, a nanoantenna or microcavity that traps light can produce strong second-harmonic signals even from materials that are only weakly nonlinear in bulk [132].

Recent studies have demonstrated highly efficient THG in engineered nanophotonic metasurfaces. A notable example is hybrid metasurfaces composed of graphene and high-index dielectrics operating in the terahertz regime. By integrating graphene layers (which have a nonlinear surface conductivity) with a dielectric metasurface, researchers achieved large THG conversion efficiencies at relatively low pump intensities. The graphene provides an ultrafast, electric-field-tunable nonlinearity, while the dielectric resonator structure (along with a metallic ground plane) induces gap-plasmon and cavity resonances that amplify the fundamental and third-harmonic fields. In one device, a bilayer graphene/dielectric metasurface achieved a THG conversion efficiency of the order of 0.32% (−24.9 dB), several orders of magnitude higher than in bare graphene, by exploiting these resonances. Such enhanced THG at terahertz frequencies is promising for applications in terahertz imaging and sources, and it demonstrates how hybrid nanomaterials can overcome the generally weak nonlinear response of monolayer graphene [133].

All-dielectric nanostructures have also shown impressive nonlinear optical results. Silicon, GaP, or LiNbO_3_ metasurfaces can be designed to support multipolar Mie resonances that overlap at fundamental and harmonic frequencies, thus boosting second-harmonic or sum-frequency generation. For example, resonant silicon metasurfaces have achieved record-high second-harmonic generation by utilizing a Fano resonance to enhance the local field. Another approach is to use epsilon near zero (ENZ) materials (materials whose permittivity approaches zero at some frequency) for nonlinear optics: at ENZ frequencies, the effective nonlinearity is greatly enhanced, and the phase matching is relaxed. ENZ-based nanostructures, such as indium tin oxide (ITO) thin films or nanostructured metamaterials at ENZ, have shown giant nonlinear index changes and frequency conversion efficiencies orders of magnitude larger than those in conventional materials. Researchers demonstrated, for example, that an ultrathin ITO film at its ENZ wavelength can generate a significant third-harmonic signal with modest pump intensities due to field enhancement inside the ENZ layer [134].

Nonlinear nanophotonics is crucial for applications like frequency comb generation on chip (using microresonators for optical frequency combs), all-optical switching (using light to control light for signal processing, leveraging intensity-dependent refractive indices), and generation of entangled photons (through spontaneous parametric down-conversion in nonlinear waveguides for quantum photonics). Ultrafast dynamics are also accessible: nanostructures can localize light to subpicosecond interaction times, enabling THz bandwidth modulators or gating of signals [135].

**Figure 4:**
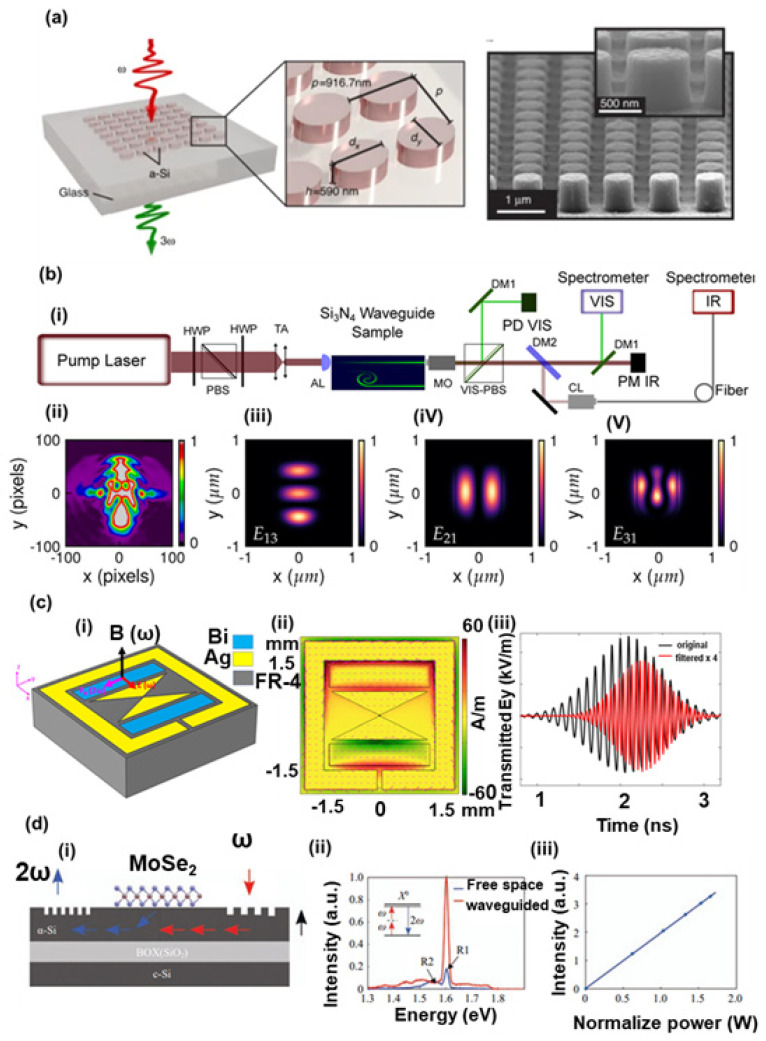
Nonlinear optical nanophotonic processes. (**a**) Metasurface for third-harmonic generation (THG): all-dielectric metasurface with design parameters for enhanced THG [137]. (**b**) Second-harmonic generation (SHG) in Si_3_N_4_ waveguides: (**i**) experimental setup; (**ii**) far-field SH intensity in the far field (CCD image); (**iii**–**v**) calculated waveguide modes (E_13_, E_21_, E_31_) for a waveguide of 0.7 × 1 µm waveguide (horizontal polarization) [138]. (**c**) Resonant nanocavity for χ^(3)^ processes: (**i**) unit cell of a SHG metasurface with split-ring resonators and bowtie nanoantennas; (**ii**) magnetic field distribution at 10 GHz; (**iii**) transmission spectrum [139]. (**d**) Hybrid on-chip nonlinear systems: SHG enhancement in (**i**–**iii**) MoSe_2_-integrated planar Si waveguides [140]. Panels (**a**–**d**) are reproduced with permission from open-access articles (refs. [137,138,139,140]).

Challenges remain in managing the heat and potential damage from high intensities in small volumes, as well as phase-matching in tiny structures (which often must rely on resonant enhancement rather than momentum conservation over a propagation length). However, the field of nonlinear nanophotonics has grown rapidly, and *metanonlinear* devices (nonlinear metasurfaces, frequency-converting metastructures, etc.) are emerging as compact optical sources (to generate new colors like UV or mid-IR on a chip) and ultrafast all-optical control elements [136]. Figure 4 illustrates nanophotonic nonlinear optical processes, including (a) a metasurface for third-harmonic generation (THG), (b) second-harmonic generation (SHG) in Si_3_N_4_ waveguides, (c) a resonant nanocavity for enhanced χ^(3)^ processes, and (d) hybrid on-chip nonlinear systems. This schematic demonstrates how nanostructures can significantly enhance nonlinear optical phenomena.

### 4.4. Photonic Integrated Circuits (PICs)

Nanophotonic materials contribute significantly to the miniaturization and performance of photonic integrated circuits (PICs), chips that integrate multiple photonic functions (light generation, routing, modulation, detection) analogously to electronic integrated circuits. Using nanophotonic waveguides and devices, PICs can achieve high component density and advanced functionality on planar substrates. Key nanomaterials and structures in PICs include high-index contrast waveguides (e.g., silicon-on-insulator wires or silicon nitride waveguides), plasmonic interconnects, and hybrid plasmon–dielectric devices [34].

One of the driving applications of PICs has been optical interconnects and data communications, where integrating many optical channels on a chip can vastly increase bandwidth while reducing size and power. High-index dielectric waveguides (such as silicon) enable tight mode confinement (mode cross sections on the order of a few hundred nm) and sharp bends, allowing dense routing of optical signals on chip [141]. Silicon photonics in particular has matured as a platform, with modulators, wavelength division multiplexing filters, and photodetectors integrated on the same silicon substrate. Recent advances in CMOS-compatible materials and fabrication methods have accelerated the integration of photonic components with electronic circuits, paving the way for energy-efficient optical computing and interconnects. For example, 800 Gbps and 1.6 Tbps optical transceiver chips are on the horizon, achieved by tightly integrating electronic drivers with dense photonic modulator and detector arrays in silicon photonics to meet the bandwidth demands of data centers [141].

Beyond pure dielectrics, plasmonic components offer the possibility of extreme miniaturization below the diffraction limit, which could be useful for interfacing PICs with nanoscale active devices or for achieving ultrasmall optical circuits. Plasmonic waveguides (e.g., metal–insulator–metal structures) can guide light in mode areas far smaller than conventional waveguides, at the cost of higher loss. Hybrid plasmonic–dielectric waveguides combine a dielectric core with a nearby metal to squeeze light slightly beyond the diffraction limit while mitigating loss, useful for compact modulators or sensors on chip [142].

A major recent milestone in PICs is the heterogeneous integration of lasers and gain media onto photonic chips. Silicon lacks an efficient light source (due to its indirect bandgap), so integrating III–V semiconductor lasers (like InP-based devices) has been a long-standing goal. In 2022, wafer-scale processes were demonstrated in which indium phosphide dies (containing multiple lasers) were bonded or transferred onto silicon wafers with prefabricated photonic circuits. This hybrid integration allows on-chip light sources to be co-packaged with modulators and other components, resulting in fully integrated transceiver chips. Intel and others achieved bonded InP/silicon laser integration using flip-chip approaches and demonstrated multiple lasers coupled into silicon waveguides across a wafer. These developments mark a turning point in making photonics as manufacturable as electronic [143].

Beyond traditional silicon and III–V materials, 2D materials are being integrated into photonic chips to introduce new functionalities: for example, graphene layers on waveguides enable electrically tunable modulators and photodetectors, enhancing broadband operation. Likewise, hybrid material approaches (combining plasmonic and dielectric components) are explored in PICs for ultracompact modulators, where a plasmonic slot adjacent to a silicon waveguide can significantly shrink device length while maintaining acceptable losses. (While 2D materials are not yet widespread in commercial PICs, they are actively researched for next-generation devices, as described above).

Another burgeoning area is using novel materials in PICs for better performance: for example, thin-film lithium niobate on insulator (LNOI) has emerged for integrated electro-optic modulators and frequency converters, offering high speed and nonlinear efficiency on chip. Two-dimensional materials such as graphene are being integrated into waveguides to create broadband optical modulators and photodetectors that can be tuned electrically [144].

PICs are also finding use in sensors and microwave photonics (e.g., integrated LiDAR systems for autonomous vehicles rely on photonic chips for beam steering and signal processing). A particularly interesting application is photonic computing and neuromorphic processors, where arrays of waveguides and optical interference units on the chip perform matrix operations at the speed of light. Demonstrations of photonic tensor cores for AI inference and coherent Ising machines for optimization have been reported, which leverage nanophotonic circuits for computation [145].

**Figure 5:**
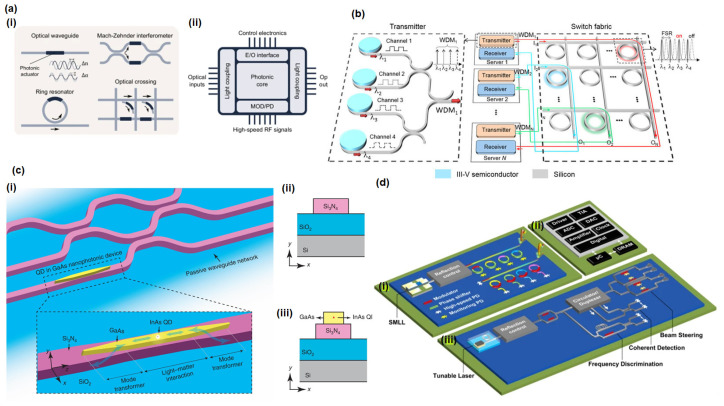
Photonic integrated circuits (PICs) enabled by nanophotonics. (**a**) Core components: (**i**) programmable photonic unit cells and actuators; (**ii**) photonic processor with optical I/O and electronic drivers [146]. (**b**) Optical interconnect: multichannel transmitters (microresonators) and a WDM crossbar switch [147]. (**c**) Quantum integration: (**i**) hybrid GaAs/Si_3_N_4_ circuit with a quantum dot (QD) for light–matter coupling; (**ii**,**iii**) cross sections of active (GaAs) and passive (Si_3_N_4_) waveguides [148]. (**d**) Applications: (**i**) WDM transceiver; (**ii**) TIA-amplified receivers; (**iii**) FMCW LIDAR with tunable lasers. Abbreviations: WDM (wavelength division multiplexing), FSR (free spectral range), TIA (transimpedance amplifier), and FMCW (frequency modulated continuous wave) [145]. Panels (**a**–**d**) are reproduced with permission from open-access articles (refs. [145,146,147,148]).

In summary, nanophotonic materials and structures are at the heart of modern PICs. They allow light to be generated, routed, and manipulated in microscopic dimensions with low loss and high speed. Advances in integration technology (bonding, 3D stacking, and foundry-scale lithography) and the adoption of new materials continue to expand PIC capabilities. As a result, we are seeing photonic chips not only in fiber-optic communications but also in emerging areas like quantum information (quantum PICs), optical signal processing, and lab-on-chip sensing. The integrated photonics ecosystem is rapidly growing, analogous to the early days of VLSI electronics, and nanophotonics are driving and benefitting from this growth [145]. Figure 5 shows some examples of photonic integrated circuits (PICs) enabled by nanophotonics. 

### 4.5. Metasurfaces and Flat Optics

Metasurfaces, composed of subwavelength nanostructures (often called ‘meta-atoms’) arranged in a carefully designed pattern on a surface, can precisely control the amplitude, phase, and polarization. They serve as extremely thin optical elements, essentially replacing conventional bulk optics (lenses, prisms, filters) with nanostructured surfaces only a few hundred nanometers thick. This capability has led to the notion of *flat optics*: optical devices with planar form factors enabled by metasurfaces [149].

A typical metasurface consists of an array of nanostructures (which can be plasmonic resonators or dielectric Mie resonators) that impart spatially varying optical responses. By engineering the geometry of each nanoelement, one can control the phase delay experienced by light passing through or reflected from that position. For example, a metalens is a metasurface that focuses light like a conventional lens but in a flat layer, by introducing a position-dependent phase profile that causes incoming wavefronts to converge to a focus. Metalenses have been demonstrated for applications from visible imaging to infrared focusing, achieving diffraction-limited performance in a much thinner package than glass lenses [150].

Metasurfaces also enable beam shaping and holography. They can be designed to produce arbitrary diffraction patterns, generating holographic images or complex beam shapes for advanced imaging systems and displays. Because they can also manipulate polarization, polarization beam splitters or waveplates can be created in the metasurface format.

Recent research in metasurfaces is increasingly using hybrid material platforms. For instance, by integrating monolayer graphene (a 2D material) with a dielectric metasurface, one can achieve dynamic tunability of the optical response via electrostatic gating. Such graphene–dielectric hybrid metasurfaces have demonstrated active control in the infrared and terahertz regimes, where the change in conductivity directly modulates the reflection or transmission. Similarly, other hybrid combinations (e.g., plasmonic nanoparticles on dielectric metasurface backbones) are employed to enhance nonlinear optical generation or to introduce gain media for loss compensation.

A significant trend in recent years has been active metasurfaces with tunable surfaces. Traditional metasurfaces are passive and have fixed functionality once they are fabricated. Active metasurfaces incorporate materials that can change their optical properties under external stimuli (electrical, thermal, optical, or mechanical). Examples of tunable components include [151,152]:

(1) Phase-change materials (eg, Ge_2_Sb_2_Te_5_, GST): These can reversibly switch between amorphous and crystalline states with vastly different refractive indices. Thus, a GST-integrated metasurface can be reconfigured between two (or more) different optical functions by a heating pulse [153].
(2) Liquid crystals: By integrating liquid crystal layers above a metasurface and applying electric fields, one can continuously tune the refractive index that the metasurface elements experience, thus adjusting the output phase pattern. This approach has been used to produce tunable focal length metalenses and beam steerers.
(3) Graphene and other 2D materials: Graphene’s optical conductivity can be electrically tuned, enabling metasurface modulation, especially in the infrared and terahertz ranges. A graphene-based metasurface can act as a dynamic mirror or filter controlled by a gate voltage [154].
(4) Microelectromechanical systems (MEMS): Some metasurfaces use MEMS actuators to physically reconfigure the meta-atom positions or orientations. For example, a series of nanomirrors on tiny actuators can tilt to steer beams (used in some LiDAR systems). Metasurfaces can also be made tunable via various mechanisms (electrical, thermal, mechanical, etc.), as discussed later in Section 5. For example, MEMS-actuated metasurface elements allow dynamic reconfiguration of the optical response (see Section 5.1 for details).
(5) Electro-Optical and Carrier Injection Tuning: Incorporating materials such as indium tin oxide (ITO) near its ENZ point, or using carrier injection in semiconductors, can modulate the refractive index of meta-atoms with nanosecond response times, acting as a high-speed spatial light modulator [155].

Active metasurfaces are crucial for emerging applications in which static optics will not suffice, such as augmented reality (AR) displays (which require dynamic holographic projection), LiDAR (which benefits from solid-state beam steering with no moving parts), and adaptive optics for imaging and communications. For example, a tunable metasurface can steer a laser beam across a wide field of view for LiDAR; one prototype leveraged a phased array of metasurface antennas controlled to scan beams at MHz speeds [156].

Another milestone was the commercial introduction of metasurface optics in consumer devices. In 2022, a partnership between the Metalenz startup and STM microelectronics led to the world’s first metasurface-enabled optical module for smartphones. Specifically, a metaoptic replaced certain lens elements in a time-of-flight 3D sensor (used for features like face ID and gesture recognition). The metasurface, manufactured on a silicon wafer in ST’s semiconductor fab, performs depth sensing by collimating and polarizing the VCSEL (laser) illumination in a very thin form factor. This marked the debut of metasurfaces in millions of consumer devices, demonstrating advantages in size (flat form), multifunctionality, and manufacturability of metaoptics for real-world applications [157].

**Figure 6:**
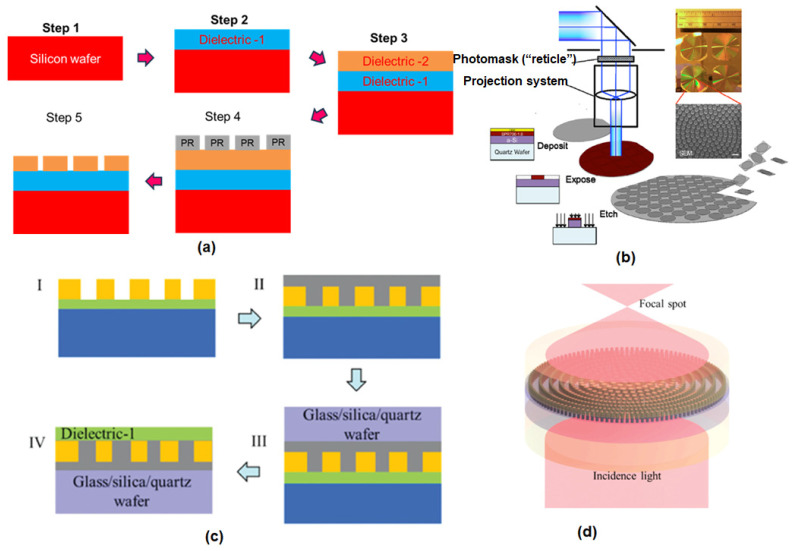
Examples of metasurface fabrication processes. (**a**) Process flow using an immersion scanner on a 12-inch Si wafer with a high-index dielectric layer [158]. (**b**) Schematic of fabricating a large-area metalens (1550 nm) including an SEM image of the nanopillar pattern at the metalens center. (**c**) Process flow to transfer a metasurface layer to a glass wafer (demonstrated on a 12-inch wafer) [158]. (**d**) Schematic of a metalens on a 12-inch glass wafer [159]. Panel (**a**,**c**) reproduced with permission from ref. [158]; panel (**b**) from ref. [160]; panels (**d**) from ref. [159].

Metasurfaces have also shown the ability to multiplex optical functions. By careful design, a single metasurface can handle multiple wavelengths or polarization states differently, acting, for example, as a lens for one polarization and a hologram for another. This multiplexing can reduce the number of components needed in optical systems. Figure 6 illustrates four distinct metasurface fabrication workflows: (a) a deep ultraviolet lithography process on a 12-inch silicon wafer with a high-index dielectric layer, (b) fabrication of large-area dielectric metalens (including an SEM inset of nanopillars) via stepwise lithography and etching, (c) a wafer-scale transfer printing technique to transplant a metasurface layer onto a glass substrate, and (d) the realization of a metalens on a glass wafer. These examples, discussed below, demonstrate how metasurfaces can be manufactured at scale using different approaches.

### 4.6. Optical Trapping and Plasmonic Tweezers

Nanophotonic structures have opened new frontiers in optical trapping by circumventing the diffraction limit of traditional optical tweezers [161,162]. Plasmonic tweezers use engineered metallic nanostructures (such as nanoapertures and nanoantennas) to create highly localized electromagnetic field gradients capable of trapping nanoscale objects, with dynamic control being a recent focus [163]. When a plasmonic nanostructure (e.g., a gold bowtie antenna or a C-shaped aperture on a metal film) is illuminated [164], it generates an intense evanescent near-field ‘hot spot’ with steep intensity gradients. Dielectric or metallic nanoparticles in the vicinity experience a strong gradient force that can pull them and confine them to these hot spots, thereby achieving trapping on the order of tens of nanometers [165].

This approach overcomes the limitations of conventional single-beam optical tweezers, which struggle to stably trap objects much smaller than ~100 nm due to weak forces at that scale. Plasmonic nanotweezers have demonstrated the capture of viruses, biomolecules, and quantum dots using modest laser powers, using field enhancements of 10^3^–10^4^× in tiny volumes [166,167]. For instance, researchers have demonstrated that a plasmonic antenna array can trap 50 nm dielectric beads using orders of magnitude less power than a diffraction-limited optical trap would require [163]. Similarly, a single plasmonic aperture in a metal film has been used to trap and manipulate individual proteins in a solution, enabling single-molecule analysis at physiological concentrations—unachievable with conventional optics.

### 4.7. Polarization-Sensitive Field Distributions

Certain nanophotonic structures create unique electromagnetic field patterns that enable specialized applications [168]. For example, a plasmonic Archimedean spiral nanoaperture generates dramatically different near-field intensity and optical force distributions depending on the handiness of the incident circular polarization [169,170]. A right-handed spiral antenna under right-circularly polarized light might concentrate energy at the spiral center, whereas left-circular polarization yields a different field topology. Such polarization-dependent responses allow these structures to function as polarization sensors or chiral molecule detectors, distinguishing input spin states via near-field signals [170,171,172].

Moreover, nanostructures can produce optical force fields with nonconservative (solenoidal) components. In a conventional optical tweezers setup, the optical force is typically a conservative gradient force in the focal region; on the contrary, near some plasmonic structures (e.g., a C-shaped aperture or spiral antenna), researchers have observed force field lines that form loops or vortices, indicating the presence of a curl (circulation) in the force field [173]. These nonconservative optical forces can induce particle rotations or complex trapping dynamics that are not possible in purely gradient-force traps. Such phenomena have been analyzed using vector field topology techniques, as demonstrated in [174]. While a full discussion is beyond our scope, we now briefly highlight that the vectorial nature of nanophotonic fields leads to rich effects (such as spin-dependent forces and topologically non-trivial field configurations) with potential uses in advanced trapping schemes and sensor devices.

### 4.8. Near-Field Scanning Optical Microscopy

An important application of nanophotonics in characterization is near-field scanning optical microscopy, which achieves super-resolution imaging by exploiting evanescent field interactions [175]. In NSOM, a tiny optical probe (typically a sharpened fiber or metallic tip with a subwavelength aperture or apex) is brought within nanometers of the surface of a sample’s surface [176]. By scanning this probe across the sample and collecting the transmitted or scattered, one can map out the optical properties (intensity, phase or spectrum) with spatial resolution far beyond the diffraction limit (often ~20–100 nm). Nanophotonic structures are central to NSOM—for example, aperture-type NSOM probes are created by coating a pulled fiber tip with aluminum except for a ~50 nm aperture at the apex, and apertureless NSOM relies on sharp plasmonic tips to localize fields. These probes confine light to nanometric volumes, allowing them to interrogate features like individual plasmonic hotspots on metasurfaces or dielectric waveguide modes in photonic circuits that conventional microscopes cannot resolve. We now mention NSOM in our review to acknowledge that near-field techniques are enabled by nanophotonics and have been crucial in probing nanostructures. In particular, NSOM has been used to directly image localized field distributions of nanophotonic devices and to validate designs by resolving details smaller than the free-space wavelength [177]. In a brief discussion of NSOM, we reinforce the connection between nanophotonic device fabrication and the tools used to characterize optical behavior at the nanoscale.

Table 3 is a comparative overview table that summarizes the five key application domains in nanophotonics: quantum photonics, biosensing, nonlinear optics, photonic integrated circuits (PICs), and metasurfaces. The table includes the focus of each domain, typical nanophotonic platforms, and representative achievements with selected references.

In summary, metasurfaces represent one of the most game-changing developments in nanophotonics, encapsulating the promise of making optical components ultrathin, planar, and multifunctional. Current research in metasurfaces not only push performance (efficiency, bandwidth) closer to that of refractive optics but also add dynamism (tunability and programmability). We anticipate that flat optics will increasingly appear in consumer products (cameras, sensors, displays) and specialized instruments, benefiting from continued progress in nanofabrication and material integration that nanophotonics provides.

## 5. Recent Advances and Trends

The nanophotonics field has witnessed transformative breakthroughs in recent years, driven by synergistic advances in materials science, fabrication technologies, and computational design. This section provides an in-depth analysis of cutting-edge developments that redefine the capabilities and applications of nanophotonic systems.

Rapid progress in nanophotonics-based nanomaterials has been driven by innovations in materials science, nanofabrication, and device engineering. This section highlights notable recent advances (primarily from the past 5 years) and emerging trends that are shaping the future of nanophotonic technologies.

### 5.1. Tunable and Active Nanophotonics

Traditional passive nanophotonic systems are increasingly being replaced or complemented by active platforms that allow dynamic control of optical properties. As discussed with active metasurfaces, the ability to tune a device’s response in real time or on demand is highly desirable. Recent advances span a variety of mechanisms and materials for tunability.

(1) Electrically Tunable Devices: Graphene, ITO, and other conductive oxides have been used to make electro-optic modulators and variable absorbers at the nanoscale. For instance, graphene integrated on plasmonic antennas can modulate mid-IR light by changing its carrier density. When operating near its ENZ frequency, ITO can function as a high-speed optical modulator, as small changes in carrier concentration induce large refractive index shifts. These approaches enable ultracompact modulators for telecommunications and dynamically tunable filters or absorbers for beam shaping [188].
(2) Phase-Change and Thermally Tunable Devices: Phase-change materials (PCMs) such as GST have been incorporated into photonic elements including metasurfaces. By toggling the PCM state, one can switch a device between two distinct optical functions (reconfigurable mirror to diffuser, lens to flat window, etc.). Although thermal tuning tends to be slower (micro to milliseconds) and requires heating, it is nonvolatile, which means the state is retained without power. Other materials such as VO_2_ (which undergoes an insulator–metal transition around 68 °C) have been used for all-optical and thermal switching in nanophotonics [189].
(3) Mechanical and MEMS Tuning: There is a resurgence of interest in nano-optomechanical systems. One example is MEMS-based metasurfaces, where nanostructures can be physically reoriented or displaced by tiny actuators. Another example is stretchable or flexible nanophotonic devices (using polymer substrates) in which mechanical stress adjusts the lattice spacing or orientation of nanostructures, thus changing their optical response [190].
(4) Nonlinear and All-Optical Tuning: Rather than external electrical control, high-intensity optical pulses can, themselves, reconfigure a nanophotonic device through nonlinear effects. Ultrafast lasers can transiently change the refractive index via the optical Kerr effect or by free carrier excitation, enabling phenomena like all-optical switching. In nanophotonic cavities, even a single or few photons can alter the resonator conditions (in the quantum regime), which is relevant for quantum photonics [191].

The collective result of these advances is a suite of tunable nanophotonic components, e.g., electro-optic metasurfaces for beam steering, graphene-based high-speed modulators, reconfigurable photonic circuits that route signals based on an applied voltage, and so forth. Such systems are crucial for adaptive optics, smart sensors, and programmable photonic processors. Indeed, the concept of programmable photonics has emerged where large arrays of tunable optical elements can be controlled via software (often drawing inspiration from phased-array radars or spatial light modulators). Examples include programmable lens arrays for dynamic focus and reconfigurable interferometer networks for on-chip signal processing.

One challenge being addressed is the speed-power trade-off: some tunable mechanisms (like thermal or MEMS) are low-power but slow, whereas others (electro-optic or all-optical) are fast but can require static power or high optical intensities. Innovative material engineering, such as new electro-optic polymers or low-loss phase-change alloys, aims to improve this. Another area of progress is integrating *feedback and intelligence* into photonic tuning, e.g., using sensors and controllers to stabilize a tunable filter automatically or using machine learning algorithms to find the configuration for a desired optical transfer function (see Section 5.4).

Overall, the trend toward active nanophotonics is enabling real-time reconfigurability in optical systems that were once static, bringing photonics closer to the electronic paradigm of versatile, programmable hardware.

### 5.2. Large-Area and Scalable Fabrication

Scaling nanophotonic devices from laboratory prototypes to industrial-scale production remains a key challenge. Many early demonstrations in nanophotonics relied on slow, small-area fabrication tools (like EBL on tiny fields). In recent years, substantial progress has been made in scalable nanofabrication techniques to bridge this gap, enabling large-area nanophotonic surfaces and high-volume manufacturing:

(1) Nanoimprint Lithography (NIL): As mentioned in Section 3, nanoimprint lithography (NIL) offers a path to high-throughput nanopatterning; here, we highlight that recent advancements in NIL have enabled wafer-scale patterning of metasurfaces with sub-50 nm features, greatly improving scalability. Likewise, deep-UV projection lithography, already capable of mass-producing nanophotonic structures, has been pushed to its limits (and complemented by emerging EUV lithography) to fabricate complex metasurface designs across entire wafers, addressing the scalability challenge. Researchers have demonstrated wafer-scale fabrication of metalenses and holographic metasurfaces using NIL, achieving feature resolutions down to tens of nanometers with excellent uniformity. Roll-to-roll nanoimprint systems now exist that can continuously print nanopatterns onto flexible films, producing meter-scale nanophotonic films (useful for, say, large-area diffractive waveplates or anticounterfeiting holograms on banknotes). A specific example is the roll-to-roll fabrication of plasmonic color metasurfaces, which was performed by continuously embossing a polymer resist with a nanograting pattern and then coating it with metal. Progress in NIL is making it possible to leave the confines of the cleanroom for production and use techniques similar to a printing press or film casting for nanophotonics [192].
(2) Soft Lithography and Self-Assembly: Soft lithography uses elastomeric stamps (often patterned by an initial top-down method) to replicate patterns through contact printing or molding. This technique has been used to create large-area nanopatterned surfaces (for example, microlens arrays or photonic crystal patterns) at low costs, albeit with a slightly lower resolution than rigid methods. Self-assembly (e.g., nanosphere lithography or block copolymers) has been improved to yield more ordered patterns over larger areas, sometimes combined with shear alignment or electric fields to improve order. These approaches are being refined to fabricate, for example, antireflection coatings composed of self-assembled nanopillars on entire wafers or plasmonic nanoparticle arrays formed by self-assembly on substrates for biosensing.
(3) Advances in Photolithography: The semiconductor industry’s push to extreme ultraviolet (EUV) lithography has incidentally benefitted nanophotonics, as commercial foundries can now pattern 10 nm-scale features on 300 mm wafers. Although expensive, this means that complex photonic circuits, including subwavelength metasurface features or photonic crystal patterns, can potentially be manufactured in large quantities using adapted CMOS processes. Additionally, multiproject wafer services for silicon photonics have made it easier for researchers to obtain chip-scale photonic devices fabricated with high quality, accelerating development of PICs (as noted in Section 4.4).
(4) Materials Integration at Scale: Scalable fabrication is not only about patterning but also about material deposition and integration on large substrates. Techniques such as sputtering and atomic layer deposition (ALD) allow the deposition of high-purity nanometer films (for example, TiO_2_ or HfO_2_ for dielectric metasurfaces) uniformly across wafers. As an example, ALD was used to create high-quality TiO_2_ metasurfaces on 4-inch wafers by depositing TiO_2_ over nanoimprinted molds and lifting off [193]. Moreover, wafer bonding techniques have scaled to bonding entire III-V wafers to silicon, or polymer films to glass, enabling heterogeneous integration at scale (mentioned earlier for laser integration).

The net outcome of these advances is that we are beginning to see nanophotonic components produced in commercially relevant quantities, such as, for instance, optical sensor chips (for biosensing or spectroscopy) that contain nanoplasmonic or ring resonator structures fabricated across a wafer. Companies have manufactured diffraction gratings and meta-optics for consumer electronics by mastering a single nanostructured surface and replicating it thousands of times via imprint. The continued focus on manufacturability is vital for nanophotonics to realize its full impact outside the lab.

### 5.3. Integration with Other Platforms

Integrating nanophotonic elements with other platforms, such as microfluidics, microelectromechanical systems (MEMS), and traditional electronics opens up new application possibilities and improves functionality in existing ones. Such cross-disciplinary integration is a rising trend:

(1) Lab-on-Chip and Microfluidics: By combining photonic circuits with microfluidic channels, one can create smart lab-on-chip systems capable of real-time, high-throughput analysis of biochemical samples. Nanophotonic sensors (ring resonators, LSPR sensors, etc.) embedded in microfluidic flow cells can detect biomarkers or chemical reactions in tiny volumes. For example, a photonic chip with a microring array was integrated into a microfluidic device to simultaneously monitor multiple analytes in a medical diagnostic panel. The synergy lies in photonics providing a set of sensitive, multiplexed detectors, while microfluidics handles sample delivery and mixing. These hybrid devices are finding use in point-of-care diagnostics, environmental toxin detection, and pharmaceutical research. Nanophotonics enhances the sensitivity and reduces the footprint of such devices, which is crucial for field-deployable sensors [194].
(2) MEMS and MOEMS (Micro-Opto-Electro-Mechanical Systems): MEMS techniques are used not only for tuning metasurfaces as mentioned, but also for integrating movable optical components like micromirrors and tunable filters on the chip. There are micro-optical benches where tiny mirrors and lenses (sometimes with nanopatterned surfaces for better performance) are assembled on a silicon MEMS chip to make miniaturized spectrometers or optical switches. The convergence of MEMS and nanophotonics yields devices like optomechanical crystals, where nanophotonic cavities are coupled to mechanical resonators, enabling ultrasensitive displacement sensors and quantum optomechanic experiments on chip [195].
(3) Electronic–Photonic Integration: We mentioned electronic–photonic cointegration in the context of PICs and tunable devices. A continuing trend is the integration of CMOS electronics (drivers, amplifiers, and digital logic) with nanophotonic devices in the same package or die. This could be heterogeneous (chip-on-chip bonding) or monolithic (fabricating photonic devices in a CMOS line alongside transistors). The result is optical systems-on-a-chip that have both the ‘brain’ (electronics for control and processing) and the ‘eyes/ears’ (photonic interfaces and sensors). For example, a fully integrated optical gyroscope chip with photonic waveguides and MEMS accelerometers together with on-chip electronics was demonstrated for signal processing, drastically reducing size compared to conventional fiber gyroscopes [196].
(4) Wearables and Flexible Photonics: Integration of photonics on to flexible substrates (polymers or thin glass) is enabling wearable photonic sensors, e.g., a nanophotonic sensor array laminated on skin to monitor pulse, blood oxygen, or glucose noninvasively using optical signals. These devices require nanostructures that can be fabricated on or transferred to flexible substrates and still function reliably. Recent work achieved flexible metasurface holograms that maintain performance under bending, as well as textile-integrated optical waveguides for sensing [197].

An example showcasing integration is a compact LiDAR module: by combining a photonic chip (with beam steering metasurface antennas) with MEMS inertial sensors (for stabilization) and electronics (for processing the returning signals), one obtains a small, robust LiDAR suitable for drones or autonomous vehicles. Another example is a point-of-care diagnostic device that integrates a nanoplasmonic biosensor chip with microfluidics and a CMOS image sensor, a complete miniaturized laboratory that can detect diseases from a drop of blood in minutes [198].

The integration of nanophotonics with diverse systems demonstrates that the field is not isolated; rather, it is increasingly central to a variety of technology platforms. This trend requires solving packaging and interfacing challenges (coupling light in/out of chips, aligning components, managing heat, etc.), but the benefits, in terms of new functionality and reduced system size, are driving significant research and development.

### 5.4. Artificial Intelligence in Nanophotonic Design

Machine learning and artificial intelligence (AI) techniques are becoming powerful tools in the design and even in the real-time operation of nanophotonic structures. The complex relationship between the geometry of a nanostructure and its optical response often makes design and optimization challenging. AI, especially deep learning, offers a way to navigate large design spaces, handle inverse design problems, and optimize device performance beyond human intuition [199].

In nanophotonic design, one common problem is the inverse design: finding a structure that yields a desired optical function (e.g., a specific spectral response or field distribution). This is inherently difficult because the design space (all possible shapes, sizes, and arrangements of nanostructures) is huge, and the relationship to optical response is nonlinear. Artificial intelligence and computational optimization methods (such as the adjoint method, genetic algorithms, and neural networks) have been applied with great success here. For example, using a neural network, one can ‘learn’ the relationship between metasurface pattern and the resulting far-field pattern. The trained network can then rapidly suggest a pattern that produces a desired holographic image, greatly speeding up the design process compared to trial-and-error or brute-force simulation [200].

Deep learning has also been used to enhance the efficiency of simulations. Surrogate models (neural network models that approximate the outcomes of computationally expensive electromagnetic simulations) can evaluate the optical response of new designs’ orders of magnitude faster than full simulations, enabling optimization algorithms to iterate quickly. A demonstration used a deep neural network to predict the spectral response of a nanopattern given its geometry, which was then embedded in an optimization loop to design a multiresonant structure for a broad flat optical response [201].

Another emerging area is the use of artificial intelligence (AI) for adaptive control of photonic systems. For example, a programmable photonic circuit with many phase shifters can have an enormous configuration space. Machine learning can tune the device by observing its output and adjusting inputs in a closed-loop manner (reinforcement learning), achieving optimal settings for a given task without requiring a precise physical model of the device. This has been explored for self-configuring optical interferometer meshes that automatically route signals or correct for fabrication errors [202].

AI is also playing a role in nanophotonic fabrication improvements. There are efforts using deep learning to correct proximity effects in lithography or to diagnose and compensate for variations in manufactured photonic circuits. For instance, an AI model can be trained on measured deviations of fabricated waveguides from design and then pre-distort future designs to cancel those deviations. This machine learning calibration can improve the reproducibility, yield, and performance of large photonic chips [203].

From a broader perspective, the introduction of artificial intelligence is leading to a paradigm shift from conventional manual or intuition-driven design to a data-driven approach in photonics engineering. Khaireh et al. (2023) comprehensively reviewed foundational deep-learning concepts and practical AI-driven optimization strategies in photonics, offering a clear workflow and design guidelines for newcomers. They compare iterative and direct neural inverse-design methods, highlighting each approach’s strengths and limitations [36]. One striking example was the design of a tiny nanophotonic structure to split equally two wavelengths of light; the AI-discovered pattern looked quite random (not something a human would likely propose), yet it outperformed traditional grating-based splitters [204].

Several particularly promising directions are emerging at the frontiers of nanophotonics research. Topological photonic circuits incorporating non-Hermitian physics have demonstrated robust light propagation immune to fabrication imperfections, with potential applications in fault-tolerant photonic quantum computing. Nonlinear topological insulators have shown enhanced frequency conversion efficiencies by more than three orders of magnitude compared to conventional nonlinear photonic crystals [205].

Quantum nanophotonics is advancing toward the integration of multiple quantum emitters in photonic crystal networks, enabling the creation of multipartite entangled states on a chip. Recent work has demonstrated 10-photon entanglement generation in nanophotonic waveguide arrays, representing a critical step toward scalable quantum information processing [106].

**Figure 7:**
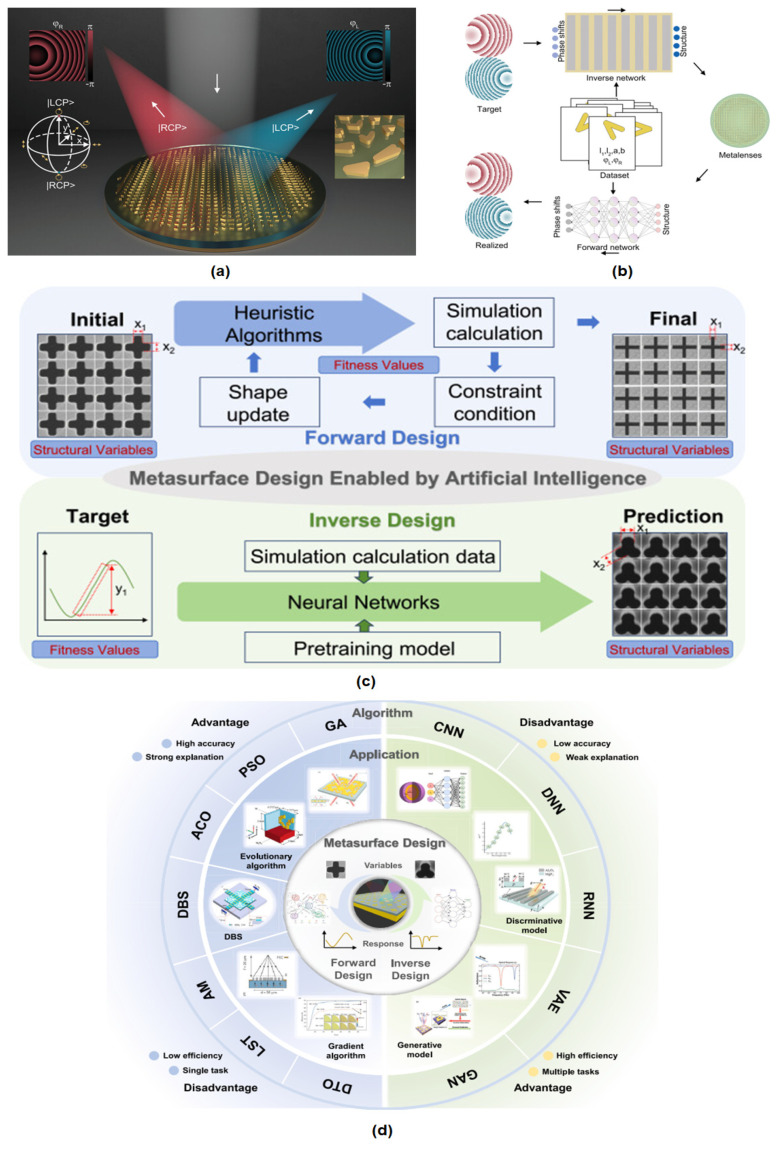
Inverse design strategies for nanophotonic structures. (**a**) Bifunctional chiral metalens operation: under normal incidence, circularly polarized light components experience distinct phase shifts, leading to separate focal points. The schematic illustrates the metasurface phase profiles for left- and right-handed polarizations, along with their representation on the Poincaré sphere. (**b**) Design workflow with deep learning-assisted design: a bidirectional neural network framework (inverse and forward models) optimizes nanostructure parameters (l_1_, l_2_, a, b) to achieve target phase responses (φ_l_, φ_r_). The inverse model generates structural designs, while the forward model validates optical performance. (**c**) Iterative AI design process for metasurfaces, integrating forward prediction and inverse optimization. (**d**) Conceptual diagram of AI-guided metasurface design, highlighting adaptive parameter tuning. Panels (**a**,**b**) reproduced with permission from ref. [206]. Panels (**c**,**d**) reproduced with permission from ref. [207].

The development of ‘intelligent’ metasurfaces that combine sensing, computation, and optical response modulation in a single thin-film platform points toward the future of autonomous optical systems. Early prototypes have demonstrated machine learning inference directly performed by nanophotonic hardware, with potential applications in ultrafast image processing and optical neural networks. Figure 7 presents a framework for AI-driven inverse design of nanophotonic structures, along with a representative device example. Specifically, panel (a) depicts the target optical functionality: a bifunctional chiral metalens that focuses left- and right-circularly polarized light to different points. Panels (b–d) then illustrate the AI-enabled design process: (b) shows a deep learning-assisted inverse design workflow (with neural networks proposing nanostructure parameters to achieve desired phase responses), (c) outlines an iterative optimization loop combining a trained inverse model and a forward model for refining metasurface designs, and (d) provides a conceptual diagram of how an AI algorithm adapts design parameters to meet performance targets. Together, these panels demonstrate how machine learning techniques can generate nanophotonic designs (such as the metalens in (a)) that meet specified optical objectives.

In summary, AI techniques are increasingly being used to predict complex optical responses, explore design spaces efficiently, and even control photonic systems in operation. This trend of ‘photonic AI’ or ‘AI-designed photonics’ is likely to accelerate, given the synergy: AI algorithms benefit from fast optical computing (an area of research itself), and photonics benefits from AI to minimize complexity. The result will be faster design cycles, improved device performance, and perhaps the discovery of novel nanophotonic phenomena that might have been overlooked through traditional design approaches [208].

## 6. Challenges

Remarkable progress in nanophotonics has brought to light several critical challenges that must be addressed to enable widespread technological adoption. This section provides a comprehensive analysis of these barriers, along with a detailed examination of innovative solutions currently being developed by the research community.

Despite significant progress in nanophotonics-based nanomaterials and devices, several challenges remain before these technologies can be widely adopted in real-world systems. This section outlines key technical barriers and provides a perspective on current efforts to overcome them.

### 6.1. Material Losses and Thermal Effects

One major limitation in plasmonic nanostructures is intrinsic material loss due to Joule heating (ohmic loss in metals), which reduces efficiency and can hinder practical device operation. Losses not only attenuate signals but also lead to significant local heating, potentially degrading device performance, or causing instability. For instance, while a plasmonic nanoantenna array can provide the necessary field enhancement for sensing applications, metal absorption may dampen the resonance and reduce its performance. Similarly, in nanoplasmonic waveguides, propagation distances are often limited to a few tens of microns or less due to loss [209].

Dielectric and hybrid materials offer lower-loss alternatives, but even they can have problems. High-index dielectrics, such as silicon or GaP, have minimal absorption loss at telecom wavelengths, but if used at visible frequencies, band-edge absorption and surface scattering can introduce loss. Two-dimensional materials such as graphene have tunable optical properties, but also inherently absorb some light (which is useful for modulators but detrimental for loss budgets in circuits) [210].

Thermal effects are closely related to losses: when nanostructures absorb optical power, they heat up, which can shift their optical response (thermal refractive index changes) or even cause physical deformation in polymers or melting in low-melting-point materials. Thermal management becomes critical, especially for high-power applications like nonlinear generation or optical computing, where many photons pass through a small volume. Strategies to mitigate these issues include using materials with better figures of merit (for plasmonics, exploring alternative materials like aluminum, which has lower loss in UV, or transparent conducting oxides at ENZ frequencies, or even superconductors for low-temperature plasmonics), and incorporating heat spreaders or thermal sinks in device design (for instance, integrating a plasmonic device on a diamond or sapphire substrate that has high thermal conductivity) [211].

Research into ENRs and high-Q resonators is one approach to mitigate losses while still achieving field enhancement. By using ENZ modes, one can achieve strong field confinement with a large effective wavelength, which can reduce scattering losses, and by engineering quasi-bound states-in-the-continuum (quasi-BICs), extremely high Q factors can be achieved even with some absorption present. For example, a carefully designed ENZ metasurface was shown to support a resonant mode with Q > 100 despite the lossy nature of the constituent material, effectively trapping light in a nonradiative state and minimizing interaction with lossy parts [212].

Ultimately, balancing loss and confinement is a pervasive challenge. In many cases there is a trade-off: tightly confined modes (especially beyond the diffraction limit) inevitably interact strongly with matter and thus incur loss, whereas low-loss modes (like in large waveguides or cavities) spread out the light more and thus have larger footprints or lower field enhancement. Finding sweet spots or clever designs (such as loss-compensated plasmonics using optical gain media or hybrid modes that confine light in a dielectric while still leveraging a metal field) is an ongoing effort.

### 6.2. Fabrication Complexity and Reproducibility

High-precision fabrication techniques, such as electron beam lithography, are excellent for prototypes but are expensive and time consuming, limiting scalability and throughput. Moreover, reproducibility across batches and manufacturing variations are concerns, especially for applications in sensitive areas such as biosensing and quantum photonics, where device-to-device consistency is paramount. A sensor array will only be as good as its worst element, and a slight variation in the size of a nanostructure could shift a resonance enough to cause errors [212].

One challenge in fabrication is the roughness of defects. Nanophotonic devices, particularly those relying on interference (ring resonators, photonic crystals), can be very sensitive to sidewall roughness or thickness variations introduced during fabrication. These imperfections cause scattering loss and resonance wavelength uncertainty. Achieving atomically smooth surfaces and edges often requires strict process control or post-fabrication smoothing techniques (such as thermal oxidation smoothing or chemical polishing for silicon waveguides) [213].

Another challenge is alignment in multistep processes. For instance, integrating 2D materials with nanophotonic cavities may require either micron-scale alignment precision during mechanical transfer or delicate electrode fabrication on graphene sheets to prevent device shorting. Each added step can introduce yield issues [214].

To combat these issues, researchers are developing more robust and forgiving design methodologies and utilizing advanced fabrication tech. Nanoimprint lithography and self-assembly have the benefit of parallel fabrication, but they must ensure uniformity; template errors in NIL can replicate across an entire wafer. Efforts are underway to perfect template fabrication and de-molding processes to minimize defects [215].

Machine learning is also being integrated into fabrication for predictive error correction. A notable example is using a neural network to compensate for the pattern layout for proximity effects and distortion in electron beam lithography. Training on known deviations (measured or simulated) can adjust the input design so that the fabricated outcome is closer to the intended one. This has improved pattern fidelity for densely packed photonic circuits, effectively acting as a smarter proximity effect correction than traditional methods [216].

In terms of reproducibility, foundry-based approaches (such as silicon photonics in commercial CMOS foundries) have helped to bring industrial process control to photonics. However, many exotic nanomaterials cannot yet be processed in those environments (due to contamination concerns, etc.), so an interim solution is modular integration, that is, fabricating nanophotonic elements separately where they can be optimized and then integrating them (e.g., via die bonding). This is performed in some cases by packaging multiple chips together (electronics + photonics) rather than a monolithic process, accepting a slight loss in compactness for a gain in yield [217].

Ensuring consistency across large areas and multiple devices is critical for commercialization. It remains a challenge, particularly for nanostructures that push the limits of dimensions. A 5% variation in diameter might be trivial in a micro-LED but disastrous in a subwavelength meta-atom. Thus, tight process control (better metrology, in-line monitoring, and feedback loops in fabrication equipment) is needed. The community is actively addressing this because without reproducibility, the leap from lab demo to real product cannot be made.

### 6.3. Environmental Stability and Durability

Nanophotonic materials often suffer from degradation as a result of environmental factors such as oxidation, humidity, UV exposure, or thermal cycling. Because nanostructures have large surface-to-volume ratios, they can be particularly vulnerable to surface chemistry changes. For example, plasmonic nanostructures made of silver initially have excellent optical properties, but silver is prone to tarnishing (sulfidation), which can severely damp its plasmon resonance over time. Similarly, bare silicon nanostructures can slowly oxidize in air, changing their refractive index and shape slightly [218].

Mechanical durability is also an issue if the nanostructures are not robustly supported. Some metasurfaces consist of very high aspect ratio pillars that could be bent or broken by slight mechanical contact or vibrations.

To ensure long-term stability, various strategies are used:

(1) Protective coatings: A thin conformal layer (a few nanometers) of a material, such as Al_2_O_3_ or SiO_2_ deposited by ALD, can seal a nanophotonic structure from the environment without significantly altering its optical function (if the coating is sufficiently thin and uniform). This is common for plasmonic structures; for example, gold nanoantennas could be coated with a ~5 nm SiO_2_ film to prevent contamination or melting into polymers [219].
(2) Encapsulation: Placing the nanophotonic devices between two glass layers or within a polymer waveguide matrix can protect them from humidity and mechanical damage. Many integrated photonic chips are now packaged similarly to electronic chips (in sealed modules) [220].
(3) Material choices: Using more inert materials is a straightforward approach. For example, choosing gold instead of silver (gold is much less reactive, though slightly more lossy at blue wavelengths), or using aluminum oxide or titanium nitride (TiN) as plasmonic materials: TiN is chemically stable and has plasmonic behavior in the visible and NIR. In dielectric metasurfaces, this means choosing materials like TiO_2_ or HfO_2_ (hard, stable ceramics) instead of something like porous silicon that can oxidize or absorb moisture [221].
(4) Ultraviolet and laser damage: High optical intensities or UV exposure can cause photoinduced changes (e.g., color centers forming in a material or ablation). Using UV-resistant materials and distributing optical power (for example, not having a single hotspot that exceeds the damage threshold) is part of a robust design. Some groups have also developed self-healing materials (e.g., certain polymers or chalcogenides that can partially reflow or reconfigure under heat to heal cracks or deformities) [222].

Long-term testing under realistic conditions (temperature cycling, humidity, etc.) is necessary to validate performance. For an optical sensor intended for the outdoors, one must ensure that it can handle temperature fluctuations and not accumulate dust or water on the surface. This introduces practical design aspects such as incorporating hydrophobic coatings to repel water or using smooth coverglass over nanostructures that can be easily cleaned [223].

In summary, while lab prototypes often operate in pristine conditions, real-world deployment requires robust engineering for stability. Nanophotonic devices are moving in that direction with the help of protective measures and smart material selection, ensuring that longevity and reliability become a strength, not a weakness, of nanophotonic technology.

### 6.4. Integration and Interconnectivity

Integrating nanophotonic components with other systems (electronics, microfluidics, and larger optical components) presents packaging and interfacing challenges. Unlike electronic signals that can be easily wired, optical signals in nanophotonics often require precise alignment with fibers or free-space optics, and maintaining low loss at these interfaces is critical [224].

Some specific challenges include

(1) Optical I/O (Input/Output): The efficient transfer of light to and off a nanophotonic chip efficiently is nontrivial. For silicon photonics, grating couplers or edge couplers are used to interface fibers to the chip. For free-space metasurfaces, one might need the metasurface carefully tilted or spaced relative to a detector or source. Misalignment of a micrometer can drastically reduce coupling efficiency. Thus, packaging often involves active alignment (tuning position while monitoring signal) and then fixing components in place with epoxy, which is time-consuming and costly. Developing self-aligned connectors or tolerantly designed couplers is a huge need. Some progress includes vertical grating couplers that have > 80% coupling efficiency with a moderate alignment tolerance, and the use of microlenses to expand and collimate beams from the chips to ease alignment [225].
(2) Electronic Integration: While we can cointegrate electronics and photonics on a chip, connecting them at high density is difficult because of thermal and crosstalk concerns. Driving many optical modulators at GHz frequencies means routing many electrical lines; these lines can heat up and also induce electrical noise. Monolithic integration (fabricating transistors and waveguides together) is challenging because processes may be incompatible (e.g., high-temperature steps for photonics can damage CMOS metals). One approach is monolithic but with process tweaks (like IBM’s CMOS-integrated silicon photonics platform, which adds photonics after transistor formation). Another is 3D integration, stacking a photonic die on an electronic die with dense microbump or even optical interconnects [226].
(3) Mechanical and Thermal Matching: Different materials have different thermal expansion coefficients. When bonding, say, an InP laser onto a silicon chip, differences in expansion can cause strain or even delamination over temperature changes. Material compatibility is important for mechanical reliability. In addition, many nanophotonic setups in the laboratory are in temperature-stabilized stages; replicating this in a product means adding thermoelectric coolers or heaters, which adds power overhead [227].
(4) Size and Form Factor: For consumer electronics or portable systems, nanophotonic components need to be integrated in a way that does not add bulk. Flat optics (metasurfaces) help reduce the form factor, but then those need to be integrated with image sensors or sources in a compact module (like Metalenz did with ST: metasurface mounted directly on the sensor package). This kind of integration, bridging the nano-optic to the device-level packaging, required developing new assembly techniques similar to how camera modules are assembled but with finer precision.

New approaches to integration include transfer printing of multiple optical components onto one substrate (pick-and-place at micrometer precision using automated tools, much like chiplet integration in electronics). Self-alignment structures are also being pursued: for example, using corner cube-shaped inserts that lock a fiber in exactly the right place when pressed on a chip edge [228].

Another concept is photonic wire bonding, where an optical waveguide is ‘written’ in 3D polymer to connect two chips optically (like an optical jumper). This has been shown to connect separate photonic dies with minimal loss, without requiring them to be on the same substrate or perfectly aligned beforehand [229].

While solutions are emerging, integration remains one of the most labor-intensive aspects of nanophotonics. There is a growing recognition that packaging can dominate the cost of photonic systems if not addressed. Therefore, the community is actively exploring techniques to simplify packaging, such as standardized photonic connectors, on-chip lasers to avoid fiber coupling, and the use of photonics for board-level or chip-to-chip links to reduce electrical pin counts (optical interconnects in computing) [230].

Machine learning-assisted fabrication control represents a paradigm shift in addressing manufacturing variability. Real-time deep learning systems integrated with electron microscopes can now detect and correct fabrication drift during the writing process, reducing feature placement errors to <0.3 nm. This capability is particularly crucial for quantum photonic circuits, where phase errors must be minimized [231].

The development of self-healing materials for nanophotonics shows particular promise. Chalcogenide glass nanocomposites have demonstrated the ability to repair radiation-induced darkening through optically stimulated atomic rearrangement, maintaining >95% transmission after exposure to 106 Gy gamma-ray exposure. Similarly, shape-memory plasmonic alloys can recover their original nanostructure after mechanical deformation through thermal activation.

In summary, achieving low-loss, reproducible interconnects, and seamless integration of nano-optics with macro-world systems are engineering hurdles. Overcoming them will likely involve both novel technical solutions and establishing standards (as electronics has performed with, e.g., USB or HDMI, one could imagine standardized optical interface chips or packages). As these pieces are put into place, nanophotonics will become far easier to deploy broadly.

## 7. Future Outlook

The field of nanophotonics stands at the threshold of a new era, where fundamental scientific advances are poised to transition into disruptive technologies that will redefine multiple industries. This section provides a comprehensive analysis of emerging frontiers and their potential social impacts, supported by recent breakthroughs and concrete development roadmaps.

Looking ahead, nanophotonics is poised for exciting developments and innovations. Several key directions are expected to shape the next era of research and technology translation:

(1) Advanced low-loss and nonlinear materials: Developing new materials with lower optical losses and higher nonlinearities is a priority. One promising avenue is the presence of near-zero (ENZ) epsilon materials, where the real part of permittivity approaches zero. ENZ materials (such as doped metal oxides or novel metamaterial composites) allow unusual light concentration and phase propagation properties, which can be harnessed for ultrastrong nonlinear interactions and tailored radiation patterns. We anticipate breakthroughs in the use of ENZ modes for ultrafast optical switching and frequency conversion at relatively low power levels. Similarly, topological photonic materials are emerging using concepts from electronic topological insulators. By designing nontrivial topology photonic band structures, one can achieve robust light transport (e.g., edge modes immune to scattering) and new localized states. Topologically protected waveguides and cavities could significantly improve device performance by reducing backscattering losses and disorder sensitivity. Already, nanophotonic topological waveguide arrays have shown the propagation of light around sharp corners with almost no loss, which is very promising for dense photonic circuits. In the coming years, we expect new material platforms (such as lithium niobate on insulation with quasiphase matching for nonlinear optics, or van der Waals heterostructures for tunable photonics) to be integrated into nanophotonic devices, combining multiple functionalities in a single device [232].
(2) Machine Learning-Driven Design and Control: The role of AI in photonics will probably expand further. Inverse design using AI will become a standard tool, and we might see the most complex photonic components (filters, couplers, modulators) being designed by algorithms that output geometries not easily conceived by humans, but which meet tight specs. This could lead to performance leaps in metrics such as bandwidth, efficiency, and footprint as devices reach theoretical limits. Moreover, AI might be embedded on chip for real-time control, giving rise to self-optimizing photonic systems. For instance, a photonic sensor network might continuously learn from its output and adjust itself (perhaps through tunable elements) to maintain optimal sensitivity or calibration. Such *intelligent photonic systems* would be highly adaptable to changing conditions, which is very useful in environments where optical properties drift (like wearable sensors subject to body temperature changes, etc.). We also foresee automated fabrication optimization: AI algorithms that adjust fabrication parameters on the fly (within advanced lithography or deposition tools) to achieve target device outcomes, reducing trial-and-error cycles [204].
(3) Programmable and Digital Metasurfaces: Closely related to AI control is the development of programmable metasurfaces, sometimes called spatial light modulators 2.0. Instead of a static metasurface performing one optical function, these would be metasurface platforms whose optical response can be dynamically reprogrammed via electronic signals (much like a field-programmable gate array in electronics). Recent research introduced the concept of digital metasurfaces where meta-atoms have two or more states (like “0” or “1”) that can be toggled to create different phase profiles. In the future, large arrays of tunable meta-atoms (perhaps MEMS-actuated or using phase-change materials) combined with electronic backplanes could yield devices that function as reconfigurable lenses, hologram projectors, or beam steering units with video rate refresh. This could revolutionize applications like AR/VR displays (enabling dynamic focal planes and focus cues), LIDAR (with no moving parts beam scanning), and optical computing (reconfiguring interconnects and weights in photonic neural networks). Achieving high pixel count, speed, and efficiency simultaneously in these dynamic metasurfaces will be a challenge, but progress in materials such as liquid crystal polymers, electro-optic polymers, and low-loss phase-change compounds provides a pathway [233].
(4) Quantum-Enhanced Nanophotonics: The intersection of nanophotonics and quantum technology will deepen. Nanophotonic devices will enhance quantum systems (as covered in Section 4.1) by enabling better single-photon sources, detectors, and compact quantum gates. However, quantum mechanics might enhance nanophotonics—for example, quantum illumination techniques use entangled photons to detect objects with lower energy than classical light would require, which could be integrated into nanoscale sensors for sensing with super-resolved or improved signal-to-noise ratios. Nanophotonics will also play a role in quantum networks, where quantum repeaters and transducers (converting photons from one wavelength to another or to other quantum systems) will likely rely on optical nanocavities and waveguides coupling to quantum memories (atoms, ions, and solid-state qubits). We anticipate revolutionary advances by integrating quantum emitters like color centers (e.g., NV or SiV centers in diamond) directly into photonic circuits for chip-scale quantum processors and secure communication nodes. As an example, one could imagine a quantum photonic sensor chip where entangled photons are generated and used to perform a measurement (like interferometric sensing) beyond classical limits, all within a nanophotonic circuit [234].
(5) Interdisciplinary and cross-scale integration: Future nanophotonic systems will increasingly bridge multiple length scales and domains. We will see more multifunctional systems that combine photonics, electronics, fluidics, and even biological components (such as embedding cells on a photonic chip for lab-on-chip diagnostics). This requires collaborative advances: materials scientists delivering better functional materials (gain media, nonlinear crystals, and flexible substrates), electrical engineers co-designing circuits to drive and read photonic devices, chemists devising robust surface chemistries for biosensors, and computer scientists developing architectures to use optical computing advantages. The convergence of these fields, aided by nanophotonics as the optical glue, could produce technologies such as ultracompact spectroscopic sensors that integrate AI on the chip to instantly interpret chemical signatures, or new medical devices that use nanophotonic endoscopes with AI image analysis to identify diseased tissue in real time [124].
(6) Commercial and Societal Impact: Finally, on the horizon is the broad commercialization of nanophotonics. The appearance of metasurface optics in consumer phones in 2022 is likely just the beginning. We expect nanophotonic components to become common in cameras (improving image quality in thinner modules), optical communications (enabling faster internet through integrated photonic transceivers), healthcare (through portable diagnostic chips and improved imaging, such as compact optical coherence tomography devices), and environmental monitoring (ubiquitous sensors for air quality, etc.). As the production volume increases, costs will decrease, creating a positive feedback loop for further adoption. Moreover, sustainability considerations may drive nanophotonics research on, for example, photonic circuits that reduce energy consumption in data centers, or nanostructured surfaces that improve solar cell absorption (leading to greener energy). Nanophotonics can contribute to energy efficiency by enabling optical analog signal processing that might complement digital electronics for tasks like AI inference, with much lower power consumption.

In conclusion, the future of nanophotonics is bright and multifaceted. We foresee a landscape where nanophotonic technologies are deeply embedded in everyday devices, infrastructure, and scientific instruments, often invisible but providing enhanced functionality and performance. Achieving this vision will require continued innovation in materials, design, and integration. Importantly, collaborative efforts across disciplines, such as physicists, engineers, chemists, computer scientists, and more, are also required to translate fundamental advances into deployable technologies. Given the rapid progress and momentum already seen, nanophotonics is on track to become a foundational technology of the 21st century, driving breakthroughs in information processing, sensing, energy, and beyond.

## 8. Conclusions

This review provided a comprehensive overview of the rapidly evolving field of nanophotonics, highlighting how advances in materials, fabrication, and device design are driving new capabilities. We surveyed core nanophotonic material platforms (plasmonic, dielectric, two-dimensional, and hybrid systems) and discussed their unique light-manipulating properties and device applications. We reviewed state-of-the-art nanofabrication techniques, top-down, bottom-up, and hybrid approaches that enable the creation of nanoscale photonic structures, as well as emerging trends like large-area nanoimprinting and additive manufacturing for nanophotonics. We explored a broad range of applications from quantum photonics and nonlinear optics to biosensing, integrated photonic circuits, and flat optics (metasurfaces), illustrating the profound impact of nanophotonics across disciplines. Recent developments in active and tunable nanophotonic devices, scalable fabrication methods, and AI-assisted photonic design were also examined, along with ongoing challenges such as optical losses, material integration, and environmental stability.

Nanophotonics is increasingly converging with microengineering and is finding its way into practical microsystems. For example, plasmonic nanostructure arrays integrated into microfluidic lab-on-chip platforms are enabling ultrarapid biosensors for point-of-care diagnostics. Likewise, MEMS-tunable metasurface devices demonstrate the fusion of microelectromechanical systems with nanophotonic functionality, allowing dynamic reconfiguration of flat optical components. These advances underscore the growing role of nanophotonics in next-generation micromachines—miniature devices and systems with unprecedented optical capabilities. As nanophotonic components become more seamlessly embedded in electronic, microfluidic, and optical microsystems, they open new avenues in computing, communications, sensing, and healthcare.

Overall, the future of nanophotonics is exceptionally promising. The synergy of innovative nanomaterials, precise fabrication techniques, and cross-disciplinary integration is accelerating the translation of laboratory breakthroughs into real-world technologies. We anticipate that nanophotonic elements will be standard in future consumer electronics, energy-efficient communication networks, advanced sensors, and medical devices. By pushing optical science to the nanoscale and integrating it with conventional microscale engineering, nanophotonics is poised to drive a new wave of microengineered systems with capabilities far beyond what is achievable today. This convergence of the nano- and micro-worlds will continue to spur discovery and innovation, solidifying nanophotonics as a foundational technology in the 21st century.

## Figures and Tables

**Table 1:** Comparison of Nanophotonic Material Platforms.

Property	Plasmonics (e.g., Au, Ag)	Dielectrics (e.g., Si, TiO_2_)	2D Materials (e.g., Graphene, TMDCs)	Hybrids (Plasmonic–Dielectric, etc.)
Loss (Q Factor)	Low (Q < 100)	High (Q > 10^3^)	Moderate (Q~10^2^)	Tunable (depends on constituents)
Tunability	Limited (static properties)	Passive (fixed properties)	High (electrostatic gating)	Dynamic (electrical/thermal control)
Example Applications	SERS substrates, sensors	Meta-optics (flat lenses)	Modulators, photodetectors	Reconfigurable optics, quantum devices

**Table 2:** The main fabrication approaches, highlighting their advantages and limitations in the context of nanophotonics.

Fabrication Approach	Examples of Techniques	Advantages	Limitations
Top-Down	EBL, FIB milling, photolithography, NIL	– Highest resolution (to ~10 nm)—Precise arbitrary patterns – Mature for prototyping (EBL) and mass production (photolithography)	– Low throughput for EBL/FIB (serial)—High equipment and mask cost – Challenging for very large areas (stitching errors)
Bottom-Up	Nanocrystal synthesis, CVD of 2D materials/nanowires, colloidal or block-copolymer self-assembly	– Atomic-scale precision in material structure (crystallinity, interfaces)—Cost-effective, scalable over large areas (self-assembly) – Enables materials inaccessible by top-down (e.g., colloidal QDs, large 2D sheets)	– Limited control over placement/pattern geometry (often periodic or random)—Reproducibility issues (batch variations) – Additional steps needed for device integration (positioning, registration)
Hybrid	Template-guided assembly, transfer printing, roll-to-roll NIL, two-photon lithography	– Combines precision with scalability (e.g., imprinting nanoscale features across a wafer)—Allows multimaterial integration on one chip (pick-and-place of nanostructures)—Enables 3D structuring (direct laser writing)	– Process complexity (multiple steps/tools)—Potential for defects at interfaces (during transfer/imprint) – Careful design needed to integrate different techniques in one workflow

**Table 3:** A comparative overview table that summarizes the five key application domains in nanophotonics.

Application Domain	Primary Focus	Typical Nanostructures	Nanophotonic Platforms	Representative Achievements	Key References
Quantum Photonics	Generation, manipulation, and detection of single photons for quantum computing, communication, and sensing	2D materials (e.g., hBN, MoS_2_), plasmonic antennas, photonic crystal cavities, quantum dots	Plasmonic–dielectric hybrid platforms, integrated photonic chips	On-chip entangled photon sources using quantum dots -Room-temperature single-photon emitters in hBN	[178] Chan et al., npj Quantum Inf 9, 49 (2023) [179] Grosso et al., Nat Commun 8, 705 (2017)
Biosensing	Label-free detection of biomolecules, viruses, DNA/RNA, proteins, etc., with high sensitivity	Plasmonic nanostructures (Au, Ag), dielectric metasurfaces, hybrid PCF platforms	SPR, LSPR, PCF-based sensors, metamaterial absorbers	-LODs in fM or pg/mL for SARS-CoV-2 detection- High-Q dielectric metasurface sensors for protein binding	[180] Alhamid et al., Saudi J Biol Sci. 2022, 29(11):103465 [181] Chen et al., Nanophotonics, 2022, 11, 4537–4549
Nonlinear Optics	Enhancement of nonlinear processes such as THG, SHG, FWM for ultrafast optics, signal processing	High-index dielectrics (Si, GaP), 2D materials (graphene, TMDs), MIM structures	Dielectric metasurfaces, plasmonic nanogaps, layered heterostructures	-THG enhancement using bilayer graphene/Si metasurfaces- ultrabroadband SHG with 2D heterostructures	[182] Chao et al., Nanomaterials, 2025, 1–13 [183] Chakraborty et al., iScience, 2022, 22, 103942
Photonic Integrated Circuits (PICs)	Miniaturization of optical components on a chip for communication, signal processing, and computing	silicon photonics, plasmonic waveguides, dielectric resonators	SOI (silicon-on-insulator), hybrid plasmonic–silicon platforms	-CMOS-compatible modulators and photodetectors- Ultracompact optical switches and routers	[184] Baumgartner et al., Optics Express, 2021, 29, 509–516 [185] Sharifipour et al., Optics Communications, 2025, 591, 132101
Metasurfaces	Tailoring light propagation, phase, polarization, and amplitude at subwavelength scales	Dielectric metasurfaces, plasmonic nanoantennas, Huygens metasurfaces	All-dielectric (TiO_2_, Si), hybrid metal– dielectric metasurfaces	-Flat lenses (metalenses) for visible and infrared-controlled beam shaping and holography	[186] Luo et al., Nanophotonics, 2022, 11, 1949–1959 [187] Chen et al., Light: Science & Applications, 2018, 7, 84

Notes: 2D Materials: widely adopted across quantum and nonlinear applications due to the strong light–matter interaction. Plasmonic Nanostructures: excellent for biosensing but often suffer from losses in PICs. Hybrid Platforms: combine plasmonic and dielectric advantages, increasingly used in cutting-edge metasurface and nonlinear optics.

## Data Availability

Not applicable.
